# Combination human umbilical cord perivascular and endothelial colony forming cell therapy for ischemic cardiac injury

**DOI:** 10.1038/s41536-023-00321-3

**Published:** 2023-08-25

**Authors:** Farwah Iqbal, Alexander Johnston, Brandon Wyse, Razieh Rabani, Poonam Mander, Banafshe Hoseini, Jun Wu, Ren-Ke Li, Andrée Gauthier-Fisher, Peter Szaraz, Clifford Librach

**Affiliations:** 1 Create Fertility Centre, Toronto, ON Canada; 2 Virginia Tech Carillion School of Medicine, Roanoke, VA USA; 3 Toronto General Research Institute (TGRI), University Health Network (UHN), Toronto, ON Canada; 4 Department of Obstetrics and Gynecology, University of Toronto, Toronto, ON Canada; 5 Institute of Medical Sciences, Department of Physiology, University of Toronto, Toronto, ON Canada; 6 Department of Obstetrics and Gynecology, Women’s College Hospital, Toronto, ON Canada

**Keywords:** Regeneration, Heart failure, Cardiac regeneration

## Abstract

Cell-based therapeutics are promising interventions to repair ischemic cardiac tissue. However, no single cell type has yet been found to be both specialized and versatile enough to heal the heart. The synergistic effects of two regenerative cell types including endothelial colony forming cells (ECFC) and first-trimester human umbilical cord perivascular cells (FTM HUCPVC) with endothelial cell and pericyte properties respectively, on angiogenic and regenerative properties were tested in a rat model of myocardial infarction (MI), in vitro tube formation and Matrigel plug assay. The combination of FTM HUCPVCs and ECFCs synergistically reduced fibrosis and cardiomyocyte apoptosis, while promoting favorable cardiac remodeling and contractility. These effects were in part mediated by ANGPT2, PDGF-β, and VEGF-C. PDGF-β signaling-dependent synergistic effects on angiogenesis were also observed in vitro and in vivo. FTM HUCPVCs and ECFCs represent a cell combination therapy for promoting and sustaining vascularization following ischemic cardiac injury.

## Introduction

Mesenchymal stromal cells, also known as “medicinal signaling cells” (MSC)^1^, are under investigation for multiple applications in regenerative and immunomodulatory medicine. Despite their in vitro differentiation potential towards both mesenchymal and non-mesenchymal lineages, MSCs do not act as stem cells in vivo but predominantly promote healing by secreting paracrine factors^2^. MSC-secreted trophic and immunomodulatory factors limit fibrosis, inhibit apoptosis, stimulate angiogenesis, recruit, and support endogenous progenitor cells, while regulating inflammation^2^. While there is some controversy in this regard, tissue-specific MSC progenitors resident in perivascular niches can co-express pericyte markers^3^. In contrast, pericytes from multiple organs may not function as MSCs^4^. A conservative description suggests that microvascular pericytes may be a subset of tissue-resident MSCs^5^. In addition to substantial characterization of tissue-specific MSCs, pre-clinical^6–12^ and clinical studies^13–18^ have shown MSC-mediated improvements in cardiac recovery and cardiac tissue remodeling in settings of ischemic cardiomyopathies.

Published studies revealed differences in the expansion potential, immunophenotype, and therapeutic potency between fetal first trimester (FTM) and full-term human umbilical cord perivascular cells (HUCPVC), each an abundant source of MSC^19^. MSCs are a promising source for allogeneic therapy due to their inherent immunomodulatory and immunoprivileged properties. MSCs lack major histocompatibility (MHC) class II and co-stimulatory molecules, and secrete immunomodulatory cytokines, chemokines, and growth factors^20^. Allogeneic MSCs are currently under investigation in randomized, double blinded, controlled clinical trials to determine therapeutic safety, adverse events, and risk for tumor development^21^. Allogeneic adipose-derived MSCs showed safety following 24 months of acute ischemic stroke^22^. The POSEIDON trial demonstrated improved functional outcomes with no significant differences in safety profiles between allogeneic and autologous MSCs^15^. Both FTM and term HUCPVCs express low levels of immunogenic MHC class I molecules (HLA-A, B, C) with greater levels of immunoprotective molecules (HLA-G) in FTM HUCPVCs. HLA-DR/DP/DQ were not detected in both sources^19^. Our group also published low HLA-A and high HLA-G expression following cardiomyocyte differentiation of FTM and term HUCPVCs^23^. In addition to immune-evasive and modulatory phenotypes, FTM HUCPVCs express high levels of pericyte-associated markers including CD146 and platelet-derived growth factor receptor beta (PDGFR-β), and display increased angiogenic activity and form pericyte-like interactions with developing vasculature in vitro compared to term HUCPVCs^19,24–26^. Angiogenesis and vasculogenesis are both required to replace damaged and dysfunctional vasculature in cardiac ischemic tissue^27,28^. In a rat model of myocardial infarction (MI), FTM HUCPVCs delivery attenuated apoptosis, promoted favorable extracellular remodeling and angiogenesis when compared to term HUCPVCs and aged bone marrow-derived MSCs (under review). Pre-delivery strategies that boost the regenerative effects of single-cell therapies for long-term healing and repair are warranted.

The concept of “next-generation cell-based therapies” has recently emerged from the understanding that a single source of therapeutic cells may not effectively target all necessary regenerative mechanisms^29,30^. The co-delivery of c-kit^+^ cardiac stem cells (CSC) and BMSCs in a rodent model of MI^31^ and in porcine models of ischemic cardiomyopathy^32–34^ demonstrated synergistic regenerative effects. The recent CONCERT-HF trial demonstrated the safety, feasibility, and efficacy of a combined autologous CSC and BMSC therapy in patients with ischemic heart failure^35^. Investigations on the combined delivery of MSCs and other regenerative cell sources including endothelial colony-forming cells (ECFC) are currently underway^36,37^. MSCs migrate to sites of ischemic injury and secrete a variety of pro-regenerative factors that stimulate endothelial cell proliferation, while subsets of ECFCs home to injured vascular sites and become incorporated into de novo vasculature^38,39^. The delivery of two cell types that are more physiologically related compared to CSC and MSC may further compensate for limitations of single cell therapies. In addition to promoting enhanced vasculogenesis and sustained perfusion following cardiac ischemic injury, a combination therapy may target other avenues of regeneration including the mitigation of apoptosis and cardiac muscle preservation.

Endothelial progenitor cells (EPC) have shown promise regarding safety, efficacy in multiple clinical trials including ischemic heart disease, pulmonary arterial hypertension, and liver disease^40,41^. Despite favorable outcomes, the use of endothelial progenitors for clinical translation has proven to be difficult due to varying isolation/manufacturing protocols, complex phenotypes, and subset populations with varying vasculogenic properties. EPCs isolated from culture expansion have been recognized as “early” or “late” based on morphology, time of culture, phenotype, and paracrine profiles^42,43^. Early EPCs (<7 days of culture) exhibit hematopoietic profiles, with a myeloid lineage and function. These cells are spindle shaped, regress before 4 weeks of culture and are CD34^+^, CD133^+^, VEGFR2^+^, CD45^+^, CD31^+^ (refs. ^42,44^). Studies show that early EPCs do not differentiate into endothelial cells and support angiogenesis primarily by paracrine signaling. Late EPCs (2 weeks of culture) now recognized as ECFCs, are potent angiogenic cells that contribute to the repair of damaged endothelium by de novo blood vessel formation in combination with paracrine activity^42^. ECFCs are cobblestone shaped, viable up to 12 weeks in culture with phenotypic profiles of VEGFR2^+^, vWF^+^, CD31^+^, Tie-2^+^, Ve-cadherin^+^ (refs. ^44,45^). Additionally, ECFCs also have immunoprivileged properties due to low expression of MHC class I molecules and no MHC class II expression. These profiles were maintained following cell differentiation and demonstrated protection against alloantibody/complement-mediated lysis and T-cell activation. This protection was preserved in acellular aortic grafts with no signs of rejection^46,47^. ECFCs have the potential for both autologous and allogeneic applications, whereas the latter is more favorable for clinical applications due to the limitations associated with manufacturing autologous aged ECFCs. For this present study, ECFCs were expanded from rat bone marrow isolates. Based on the time of culture (2 weeks), morphology (cobblestone), and immunophenotype (Ve-cadherin^+^, VEGFR2^+^, CD133^+^, CD34^+^, CD31^-^), ECFCs with “late” EPC characteristics were co-injected with FTM HUCPVCs in the ischemic rat myocardium.

This study characterizes the efficacy of a combination cell therapy including ECFCs with de novo vascularization potential and FTM HUCPVCs, which reside in perivascular regions of the umbilical cord in an immunocompromised rat model of MI. The combination cell delivery of xenogeneic (human) FTM HUCPVCs and allogeneic ECFCs, improved cardiac function by 16% compared to FTM HUCPVC single treatments and 139% to the media injection group at 4 weeks. Decreased apoptosis, improvement in cardiac vascularization, and cardiac motility were observed in combination treatment groups. Characterization of both cell types identified pericyte-endothelial interactions mediated through PDGF-β signaling, leading to the development of perfused vasculature in the left ventricle (LV) and in angiogenesis assays. This study highlights a combination therapy which protects the ischemic myocardium at least in part via multiple interconnected angiogenic mechanisms, leading to greater cardiac recovery compared to single-cell treatments.

## Results

### Co-injected FTM HUCPVCs and ECFCs home to perivascular regions in the ischemic myocardium and show early protection of cardiac function (5 days)

**Fig. 1:**
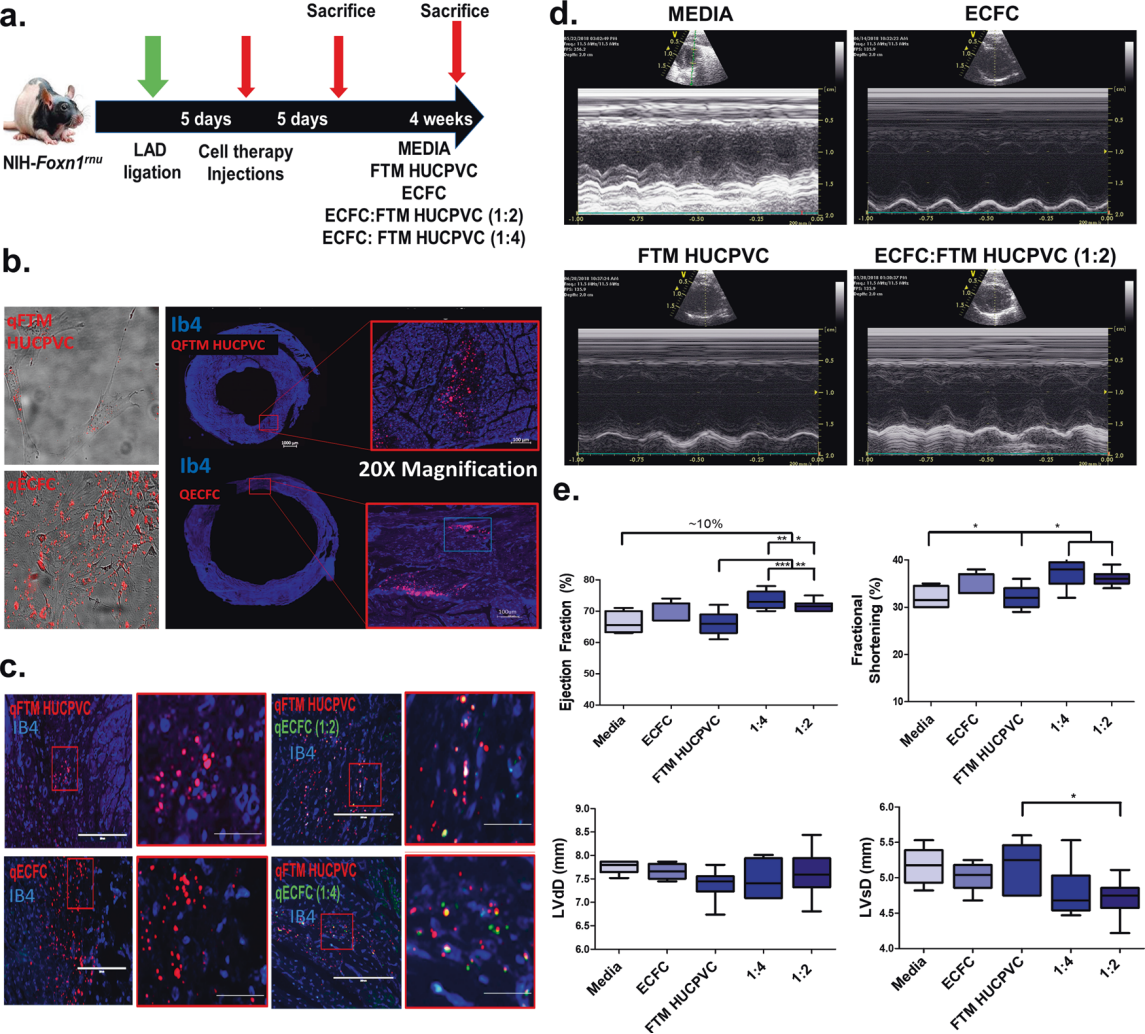
FTM HUCPVCs and ECFCs home to perivascular regions and show early cardiac benefit at day 5 post-treatment. **a** Schematic of in vivo study design. NIH Foxn1^rnu^ rats underwent left anterior descending (LAD) ligation and were injected at three sites surrounding infarct. Cell therapy included medium, single, and combination therapies of FTM HUCPVCs and ECFCs, 5-days post myocardial infarction (MI). Cardiac tissue was harvested at 5 days and at 4 weeks post injection. **b** Pre-labeled FTM HUCPVCs and ECFCs with Q-dot nanocrystals (red) prior to injection (left panel). FTM HUCPVCs and ECFCs (red) detected in the LV of infarcted hearts (right panels) 5-days following single cell injections in conjunction with Isolectin B4 (IB4, blue). Scale bar: 100 μm. *N* = 3 animals per condition. **c** Fluorescence imaging of single and combination injections of FTM HUCPVC (red) and ECFC (green) in infarcted hearts. Co-injected FTM HUCPVCs and ECFCs were co-localized (yellow signal) and were identified near capillaries labeled by IB4 (blue). Scale bar for low magnification images: 400 μm; high magnification images: 200 μm. *N* = 3 animals per condition. **d** Representative echocardiographs for all treatment groups 5-days following cell therapy. **e** Quantification of output measures (ejection fraction, fractional shortening) and left ventricular systolic dimensions (LVdD (LV diastolic dimension), LVsD (LV systolic ejection)) *N* = 5–12 animals per experimental condition. Statistical analysis by One-Way ANOVA using Tukey post-hoc test. *p < 0.05, *p < 0.01, ***p < 0.001. Data are representative of 10 fields/animal. Box plots: the black center line denotes the median value (50th percentile), while the black box contains the 25th to 75th percentiles of dataset. The black whiskers mark the 5th and 95th percentiles.

MI was induced by ligating the left anterior descending artery (LAD) in Foxn1^rnu^ immunodeficient rats. Animals were killed at day-5 post-injection and the LV was assessed for cell engraftment, apoptosis, preservation of cardiac muscle, and paracrine mechanisms. Animals were killed at 4 weeks to evaluate cardiac function, LV fibrosis, and cardiac perfusion (Fig. 1a). Fluorescently labeled FTM HUCPVCs and ECFCs were identified in the LV and co-localized near Isolectin B4^+^ (IB4) blood vessels (Fig. 1b, c). Echocardiography measurements (Fig. 1d) showed that both 1:2 and 1:4 combination groups improved ejection fraction and fractional shortening compared to FTM HUCPVC treatment (*p* < 0.05) and medium control groups (*p* < 0.02; Fig. 1e). Decreased left ventricular systolic dimensions in the 1:2 combination group were measured compared to FTM HUCPVC single treatments (*p* < 0.05; Fig. 1e). Dosing studies showed that lower numbers of injected FTM HUCPVC have greater cell retention in cardiac muscle (Supplementary Fig. 1).

### Co-injected FTM HUCPVCs and ECFCs preserve cardiac muscle by decreasing apoptosis and increasing vascularization (5 days)

**Fig. 2:**
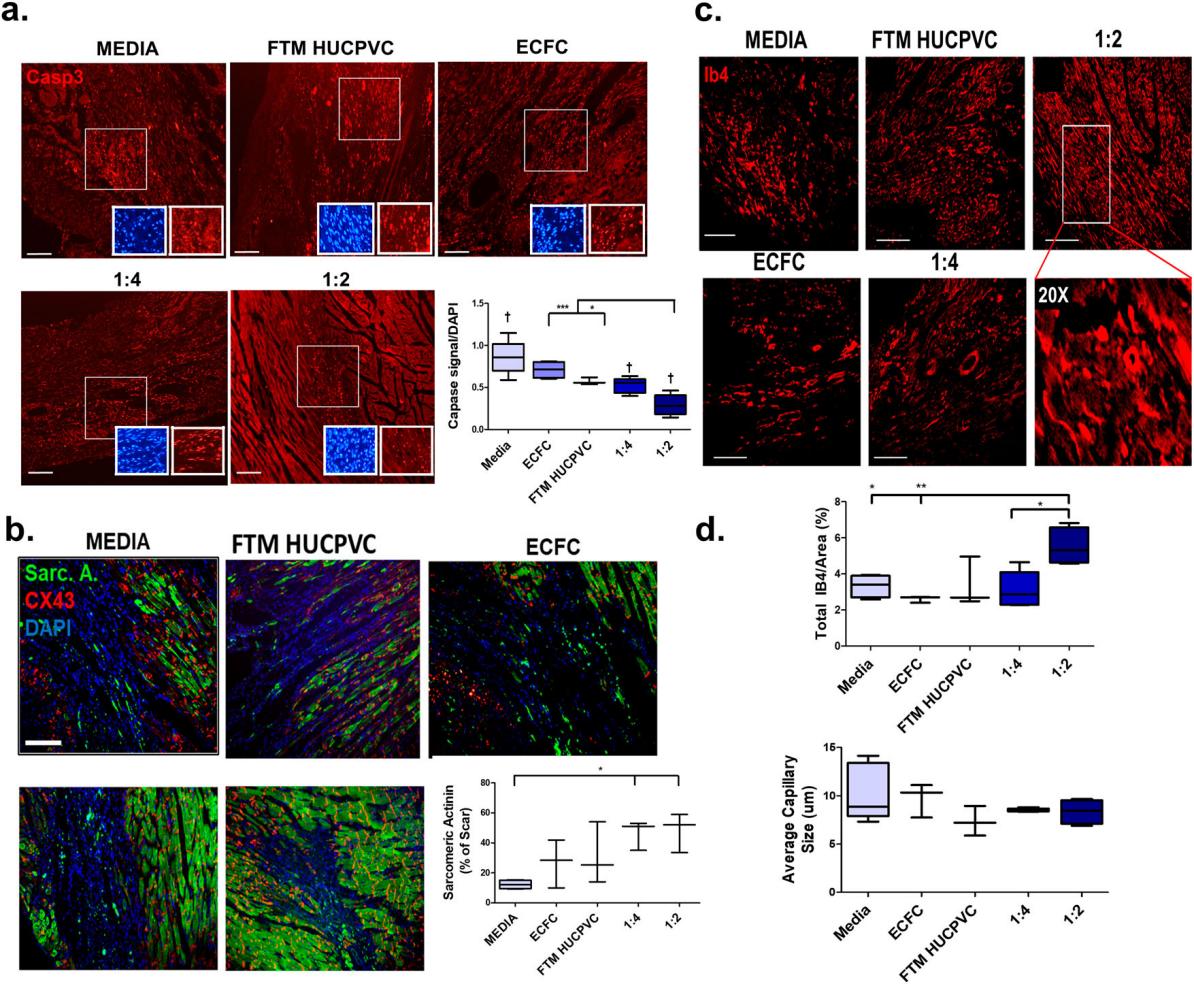
FTM HUCPVCs and ECFCs reduce cardiac apoptosis, preserve cardiac muscle and show early angiogenesis. **a** Detection of apoptosis in post-MI myocardium 5 days following cell injection. Heart sections were stained for cleaved caspase-3 (red). Apoptotic cells were quantified and expressed as a percent of total image cell nuclei (DAPI/ field). Scale bar: 200 μm. († difference from medium control, *p* < 0.001). *N* = 3–6 animals per condition. **b** Merged fluorescence images of sarcomeric actinin (green) and connexin-43 (Cx-43) (red) immunostaining of cardiac tissue sections 5-days following cell treatment. Quantification of sarcomeric actinin and Cx-43 signal density within the LV of all treatment groups, normalized to scar area. Scale bar: 200 μm. *N* = 3–4 animals per condition. **c** Fluorescence imaging and (**d**) quantification of total capillaries/field and average size of capillaries following cell treatment. Scale bar: 200 μm. *N* = 3–5 animals per condition. Statistical analysis by One-Way ANOVA using Tukey post-hoc test. *p < 0.05, *p < 0.01, ***p < 0.001. Data are representative of 10 fields/animal. Box plots: the black center line denotes the median value (50th percentile), while the black box contains the 25th to 75th percentiles of dataset. The black whiskers mark the 5th and 95th percentiles.

At 5 days, the 1:2 combination treatment group showed reduced cleaved caspase-3 positive nuclei when compared to FTM HUCPVC alone (*p* < 0.05), ECFC alone (*p* < 0.001), and medium control (*p* < 0.001) (Fig. 2a). Combination treated hearts displayed higher levels of sarcomeric actinin (Sarc. A) compared to the medium control (*p* < 0.01) (Fig. 2b). Quantification of IB4+ blood vessels (Fig. 2c) showed increased number of vessels in the 1:2 combination group compared to the 1:4 (*p* < 0.05), ECFC single injection (*p* < 0.01) and medium control groups (*p* < 0.05) (Fig. 2d). No differences in capillary size were detected. PDGFR-β^+^ FTM HUCPVCs were identified co-localized with rat CD31^+^ antigen in the LV (Supplementary Fig. 2). Collectively, decreased apoptosis and preservation of cardiac muscle were observed in FTM HUCPVC and ECFC combination treatment groups leading to early improvements in cardiac contractility.

### FTM HUCPVCs secrete pro-angiogenic factors in the left ventricle (5 days)

**Fig. 3:**
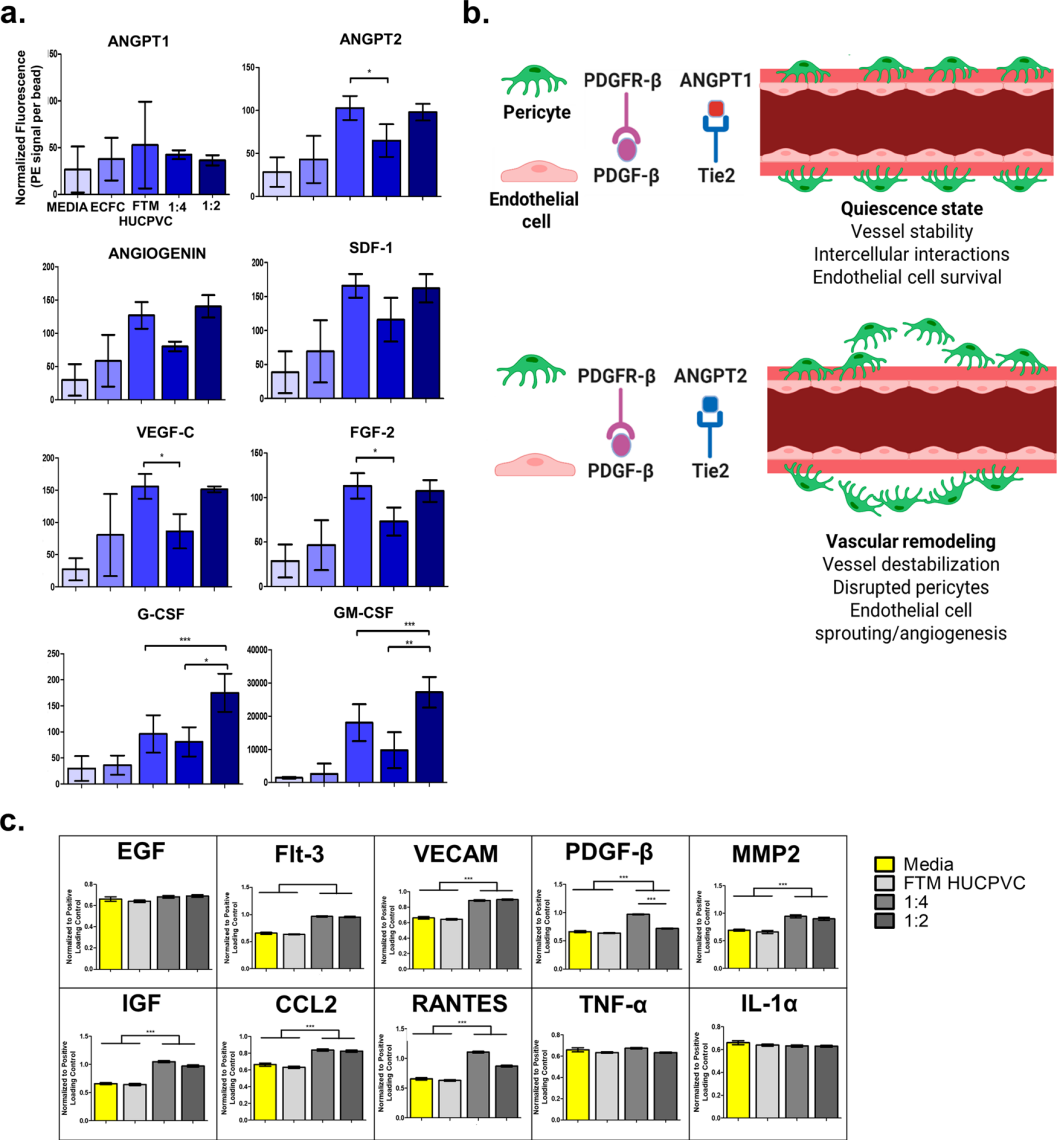
FTM HUCPVCs co-injected with ECFCs (1:2) secrete similar levels of angiogenic growth factors in the rat myocardium despite fewer FTM HUCPVCs. **a** Quantification of human pro-angiogenic factors detected in rat left ventricular tissue lysates, 5-days following cell treatments using Luminex bead assay. Conjugated beads were processed using flow cytometry, where signal was normalized to fluorescence signal per bead. Trends of increased pro-angiogenic factors were detected in both FTM HUCPVC single and 1:2 combination treatment groups compared to 1:4 cell combination, medium, and ECFC treatment groups. ANGPT2 was detected in higher levels in both 1:2 combination and FTM HUCPVC only treatment group. *N* = 3. **b** Illustrations describing the role of both ANGPT1 and ANGPT2 in tissue re-vascularization and homeostasis. **c** Quantification of host (rat) pro-angiogenic and pro-inflammatory factors detected in left ventricular lysates using rat-specific antibodies. Greater levels of Flt-3, VECAM, PDGF-B, MMP2, and IGF were detected in rat myocardium following treatment with FTM HUCPVC-ECFC cell combinations compared to medium and FTM HUCPVC single treatments (*p* < 0.001). Increases in CCL2 and RANTES were detected in combination treatment groups compared to medium and FTM HUCPVC treatment groups (*p* < 0.001), with no differences in TNF-α and IL-1α. *N* = 3. Normalized to positive loading control. Statistical analysis by One-Way ANOVA using Tukey post-hoc analysis. *p < 0.05, *p < 0.01, ***p < 0.001. Bar graphs: Data are presented as mean ± SEM.

FTM HUCPVC-secreted factors were detected in the LV using a custom human multiplex proteome panel. Both FTM HUCPVC alone and the 1:2 combination treatment groups showed similar levels of ANGPT2, ANG, VEGF-C, FGF-2, and SDF-1 in the LV (Fig. 3a). Although the 1:2 combination group contained ~66% fewer FTM HUCPVCs compared to the FTM HUCPVC single treatment, similar levels of pro-angiogenic factors were detected. Schematics show the role of ANGPT1 and ANGPT2, where ANGPT2 is required for vascular remodeling (Fig. 3b). Rat-specific ELISA were used to detect rat-secreted (injected ECFCs and host cells) factors (Fig. 3c). Increased secretion of rat-derived angiogenic growth factors (Flt-3, VECAM, PDGF-β, and IGF-1) in the myocardium were detected in combination treatment groups compared to medium and FTM HUCPVC single groups (*p* < 0.001) (Fig. 3c). Additional analysis of ECFC and FTM HUCPVC-secreted immunomodulatory and angiogenic factors can be found in Supplementary Fig. 3. Both FTM HUCPVCs and ECFCs are a potent source of paracrine factors required for vascularization, which induce the secretion of regenerative factors in the rat myocardium.

### Co-delivery of FTM HUCPVCs and ECFCs improves cardiac function compared to single-cell treatments and decreases cardiac fibrosis (4 weeks)

**Fig. 4:**
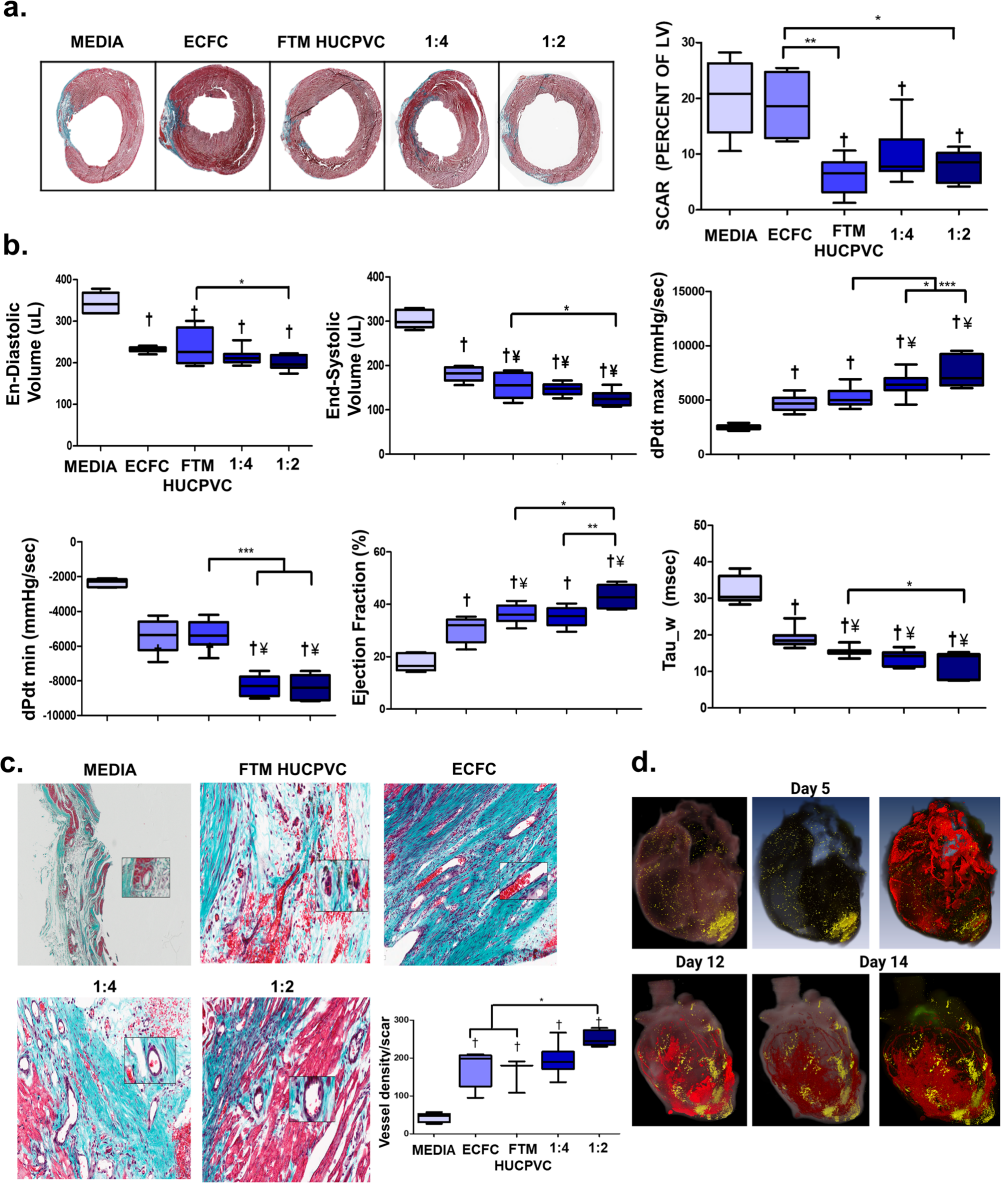
Combined cell treatments of FTM HUCPVCs and ECFCs preserve cardiac muscle and improve cardiac function at 4 weeks. **a** Representative images of Masson’s Trichrome staining of the LV and corresponding quantification of scar size as a proportion of the LV (LV). *N* = 6–9 animals per condition. **b** Pressure volume catheterization at 4 weeks following cell administration and quantification of pressure buildup and volume output measures. († significant difference from medium control, *p* < 0.001, ¥ significant difference from ECFC single injection, *p* < 0.001). *N* = 6–9 animals per condition. **c** Masson’s trichrome staining micrographs depicting left ventricular scar (blue) cardiac muscle (red) and cell nuclei (purple) with quantification of perfused vasculature. Total vasculature was quantified in scar tissue of each stained heart section. († significant difference from medium control, *p* < 0.001). *N* = 3–6 animals per condition. **d** Pre-labeled FTM HUCPVCs and ECFCs were identified using fluorescence microscopy (BioInVision) at day 5, 12, and 14. Shown in yellow are Q-dot labeled FTM HUVPVCs and ECFCs. Shown in red are the main vessels/red features in the heart volume. *N* = 3 animals per condition. Statistical analysis by One-Way ANOVA using Tukey post-hoc test. *p < 0.05, *p < 0.01, ***p < 0.001. Data are representative of 10 fields of LV. Boxplots: the black center line denotes the median value (50th percentile), while the black box contains the 25th to 75th percentiles of dataset. The black whiskers mark the 5th and 95th percentiles.

Masson’s trichrome staining identified decreased fibrosis and left ventricular scar size in combination groups and FTM HUCPVC single group, compared to ECFCs alone (*p* < 0.05) and medium control (*p* < 0.001) (Fig. 4a). Pressure-volume catheterization was performed 4 weeks following cell administration (Fig. 4b and Supplementary Fig. 4). All cell treatments led to improvements in haemodynamic functions relative to medium injections (*p* < 0.001). The 1:2 combination treatment decreased both end-diastolic and systolic volumes compared to FTM HUCPVCs alone (*p* < 0.05) and ECFCs alone (¥) (*p* < 0.01). Both combination groups significantly increased dPdt max, decreased dPdt min and decreased Tau_w, compared to FTM HUCPVCs alone (*p* < 0.001) and ECFCs alone (*p* < 0.001). Ejection fraction measurements in the 1:2 combination group were significantly increased compared to the 1:4 combination group (*p* < 0.01), FTM HUCPVCs alone (*p* < 0.05), ECFC alone (¥) (*p* < 0.001) and medium control (†) (*p* < 0.001) group (Fig. 4b). 1:2 combination treated hearts had increased perfused blood vessel density within the scar tissue compared to FTM HUCPVCs, ECFC single injections (*p* < 0.05), and medium control (*p* < 0.001) (Fig. 4c). Engrafted cells (yellow) were visualized at day 5, 12, and 14 around vasculature (red) using BioInVision technology (Fig. 4d).

### Co-delivery of FTM HUCPVCs and ECFCs increases vascularization and ECM remodeling at 4 weeks

**Fig. 5:**
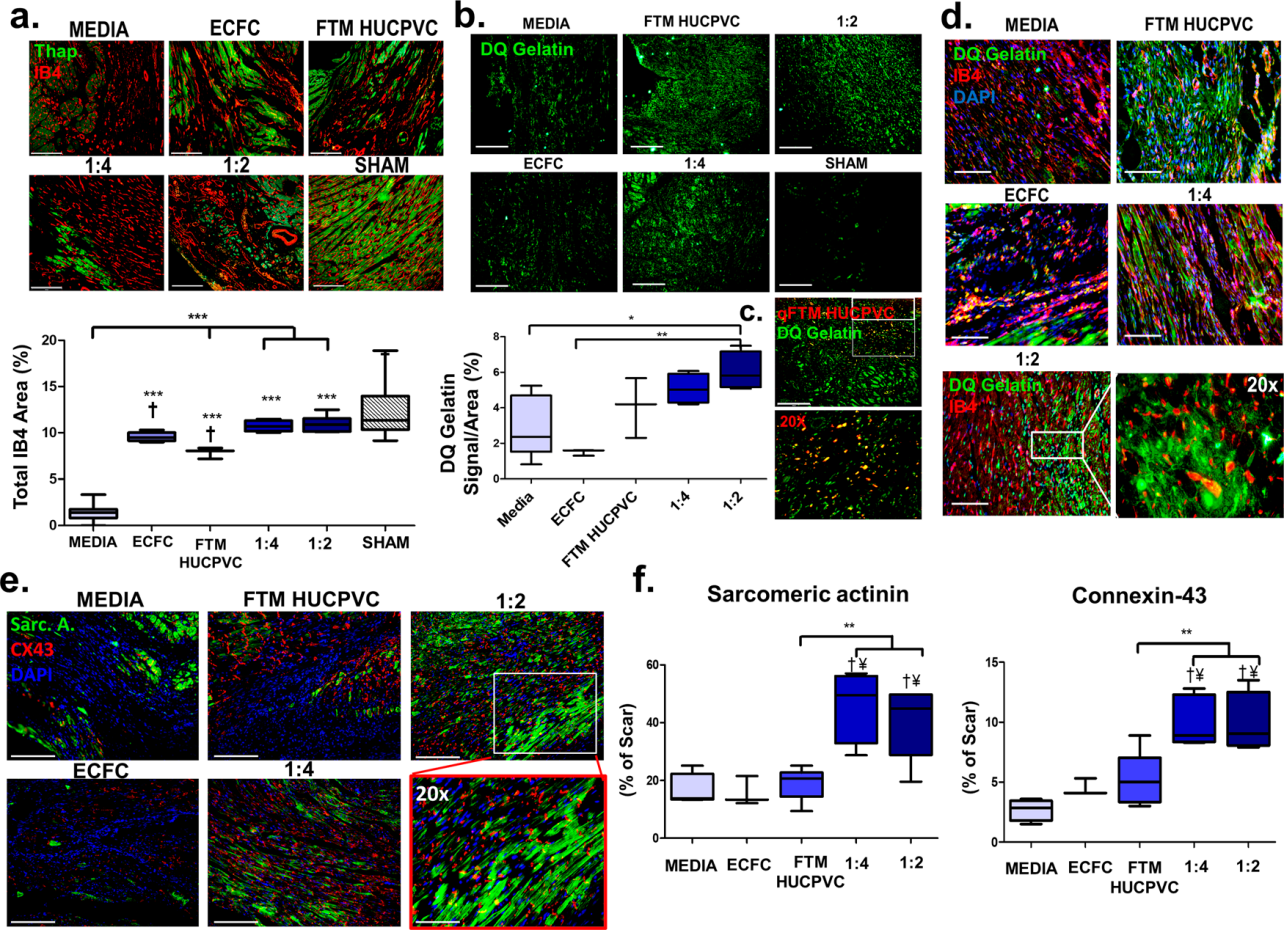
Combined cell treatments of FTM HUCPVCs and ECFCs preserve cardiac muscle by increasing ECM remodeling and angiogenesis at 4 weeks. **a** Fluorescence overlay images of IB4 (red) and counterstain thapsigargin (green). Quantification of blood vessel area (% IB4) per area. (†significant difference from medium control, *p* < 0.001, and asterisks above bars represent significant difference from unaffected cardiac tissue). The unaffected group represents quantification of vasculature from healthy LV without injury or cell treatment. Scale: 200 μm. *N* = 4–9 animals per condition. **b** ECM remodeling activity was detected using fluorogenic protease substrate DQ gelatin and signal quantified per area (%) Scale bar: 200 μm. *N* = 4–9 animals per condition. **c** Fluorescence imaging of DQ gelatin (green) and Q-dot-labeled FTM HUCPVC signal (red). Yellow signal marks overlapping signal of processed DQ gelatin and pre-labeled FTM HUCPVCs. Scale bar: 200 μm. *N* = 3. **d** Merged fluorescence microscopy images of DQ gelatin (green), IB4 (red) signal and DAPI (blue). Scale bar: 100 μm. *N* = 4–9 animals per condition. **e** Merged fluorescence images of sarcomeric actinin (green) and connexin-43 (Cx-43) (red) staining 4 weeks after cell implantation. **f** Quantification of sarcomeric actinin and Cx-43 within the LV of all treatment groups normalized to imaged scar area. (¥ significant difference from ECFC control, *p* < 0.001 and †significant differences from medium control group, *p* < 0.001). Scale bar: 200 μm. *N* = 3–6 animals per condition. Statistical analysis by One-Way ANOVA using Tukey post-hoc test. *p < 0.05, *p < 0.01, ***p < 0.001. Data are representative of 10 fields of LV. Boxplots: the black center line denotes the median value (50th percentile), while the black box contains the 25th to 75th percentiles of dataset. The black whiskers mark the 5th and 95th percentiles.

Increased IB4 signals were detected in both combination treatment groups compared to FTM HUCPVC alone and medium control groups (*p* < 0.001; Fig. 5a). Increased ECM remodeling (DQ gelatin signal) was identified in 1:2 combination treatments compared to ECFC (*p* < 0.01) and medium control (*p* < 0.05; Fig. 5b). Q dot labeled FTM HUCPVCs were identified within regions of the DQ gelatin signal (Fig. 5c). Merged fluorescence images of DQ gelatin and vascular structures (IB4), showed co-localization of both signals especially in the 1:2 combination treatment groups (Fig. 5d). Increased Sarc. A and Cx-43 levels were detected in both combination treatment groups, compared to FTM HUCPVC-single treatments (*p* < 0.01), ECFC-single treatments (¥) (*p* < 0.001) and medium control groups (†) (*p* < 0.001; Fig. 5e, f). The combination of FTM HUCPVCs and ECFCs improved cardiac function following MI by increasing vascularization and ECM remodeling, leading to the preservation of cardiac muscle and decreased fibrosis.

### FTM HUCPVCs and ECFCs initiate in vitro tube networks, where FTM HUCPVCs display pericyte interactions and secrete angiogenic growth factors

Both FTM and term HUCPVCs are a rich source of MSCs and were assessed for their angiogenic properties. The comparison of various MSC characteristics between FTM and term HUCPVCs was published previously^19,23,48^. Phenotypic characterization of FTM HUCPVCs can be found in Supplementary Figs. 5 and 6. Phenotypic characterization of ECFCs found in the Supplementary Figs. 7 and 8.

**Fig. 6:**
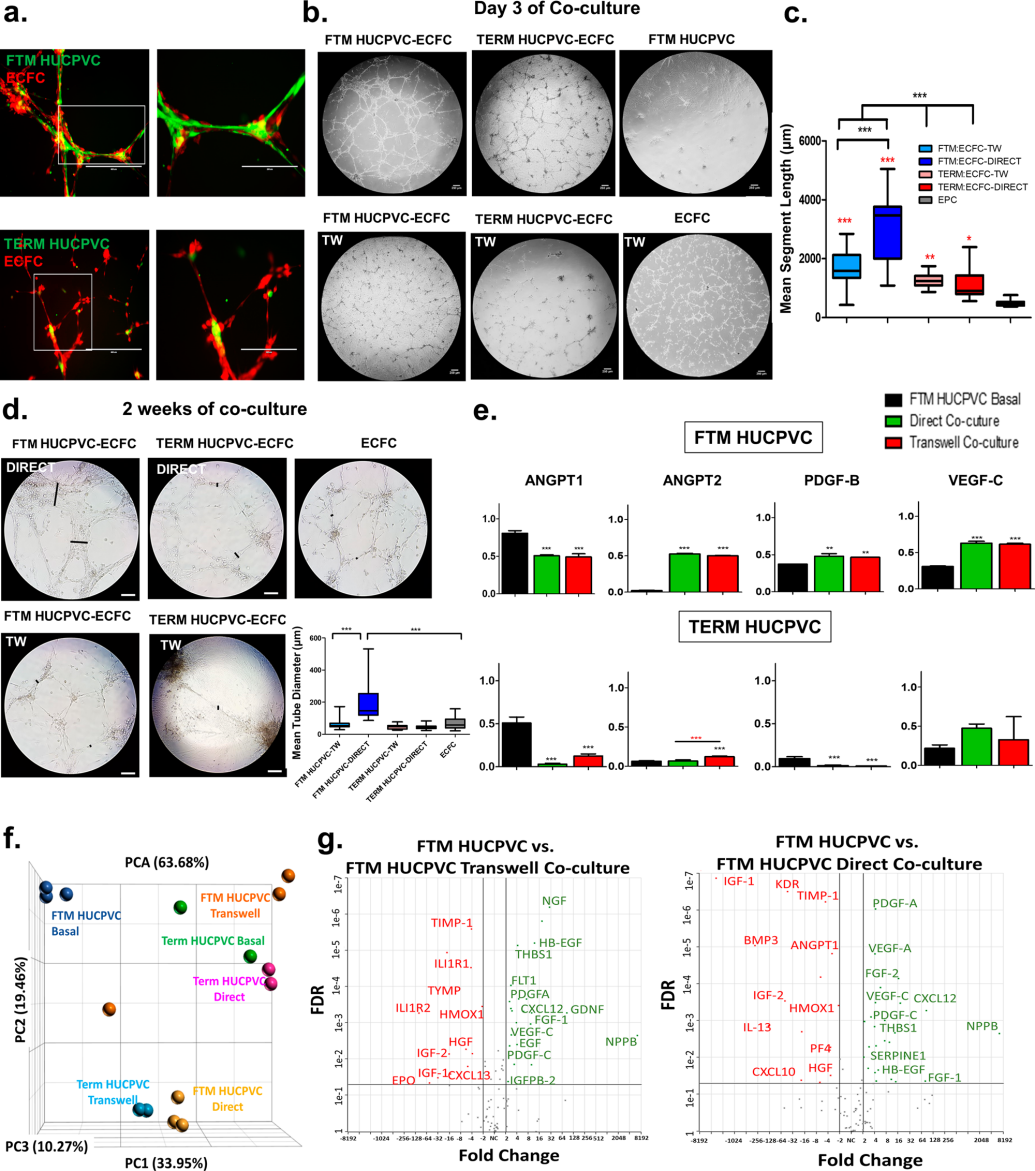
Directly co-cultured FTM HUCPVCs and ECFCs exhibit synergistic vascular potential compared to single cells in vitro. **a** Fluorescence images of pre-stained HUCPVCs and ECFCs in direct co-culture. Scale bar: 400 μm. **b** Bright field images of endothelial networks on day 3 of co-culture, with robust networks observed in direct co-culture of FTM HUCPVCs and ECFCs. Scale bar: 250μm. **c** Quantification of tube length following co-culture, with increased tubule lengths in FTM HUCPVC co-cultures (*p* < 0.001). Red asterisks represent significant differences between treatment groups and ECFC only control group (gray bar). *N* = 5 separate experiments with different donors, *n* = 17 fields of sub-analysis. **d** Bright field images of HUCPVC and ECFC-derived networks at 2 weeks following co-culture. Scale bar: 250 μm. *N* = 5 separate experiments with different donor lines, *n* = 18 fields of sub-analysis **e** Human protein array analysis of conditioned medium collected at 24 hours from FTM and TERM HUCPVC-ECFC co-cultures (direct co-cultures (green bar) transwell co-cultures (red bar) compared to basal FTM HUCPVC conditioned medium (black bar). FTM HUCPVCs secrete higher levels of angiogenic growth factors compared to term HUCPVC in ECFC co-cultures with no major differences between direct and transwell co-culture. *N* = 5. **f** Principal component analysis depicting transcriptome differences between HUCPVC treatment groups. **g** Next-generation sequencing analysis represented by volcano plots, display differential gene expression of FTM HUCPVCs following co-culture compared to FTM HUCPVCs from basal culture conditions. Statistical analysis by One-Way ANOVA using Tukey post-hoc test. *p < 0.05, *p < 0.01, ***p < 0.001. *N* = 3. Panel **c** and **d** box plots: the black center line denotes the median value (50th percentile), while the black box contains the 25th to 75th percentiles of dataset. The black whiskers mark the 5th and 95th percentiles. Panel e bar graphs: Data are presented as mean ± SEM.

HUCPVCs were either directly cultured with ECFCs on Matrigel-coated wells or cultured on transwell inserts above ECFCs to investigate paracrine only mediated effects (Supplementary Fig. 9). FTM HUCPVCs extended membrane projections between endothelial network junctions whereas term HUCPVCs expressed limited elongated morphologies 24-h following co-culture (Fig. 6a). Directly co-cultured FTM HUCPVCs and ECFCs developed continuous networks compared to term HUCPVC direct (*p* < 0.001) and transwell cultures (*p* < 0.01) at day 3 (Fig. 6b, c). At 2 weeks, the direct co-culture of FTM HUCPVCs and ECFCs led to the formation of thicker networks compared to term HUCPVC direct and transwell co-cultures (*p* < 0.001; Fig. 6d). These results suggest the need for direct co-cultures of FTM HUCPVCs and ECFCs for synergistic effects on de novo in vitro tube formation.

Conditioned media (CM) collected at 24-h post co-culture from combination cultures contained increased ANGPT2, PDGF-β, and VEGF-C levels compared to media analyzed from FTM HUCPVCs alone (*p* < 0.01) (Fig. 6e). Lower levels of angiogenic growth factors were detected in term HUCPVC cultures (Fig. 6e). See dot plots and ECFC-secreted angiogenic factors in Supplementary Figs. 10 and 11. FTM HUCPVCs and ECFCs were isolated from co-cultures at 2-weeks and processed for next-generation sequencing using human-specific primers. PCA plots show variability in the transcriptome of cell treatments (Fig. 6f). FTM HUCVPCs increased the expression of angiogenic transcripts in both direct and transwell co-cultures, including isoforms of PDGF, FGF, VEGF, and EGF (Fig. 6g). Similar increases in angiogenic transcripts by term HUCPVCs were not observed (Supplementary Fig. 12). These results suggest that while similar angiogenic factors were detected in FTM HUCPVC transwell and direct co-cultures, the direct physical contact between FTM HUCPVCs and ECFCs is required for tube formation.

### The co-injection of FTM HUCPVCs and ECFCs in Matrigel plugs promotes de novo vascularization

**Fig. 7:**
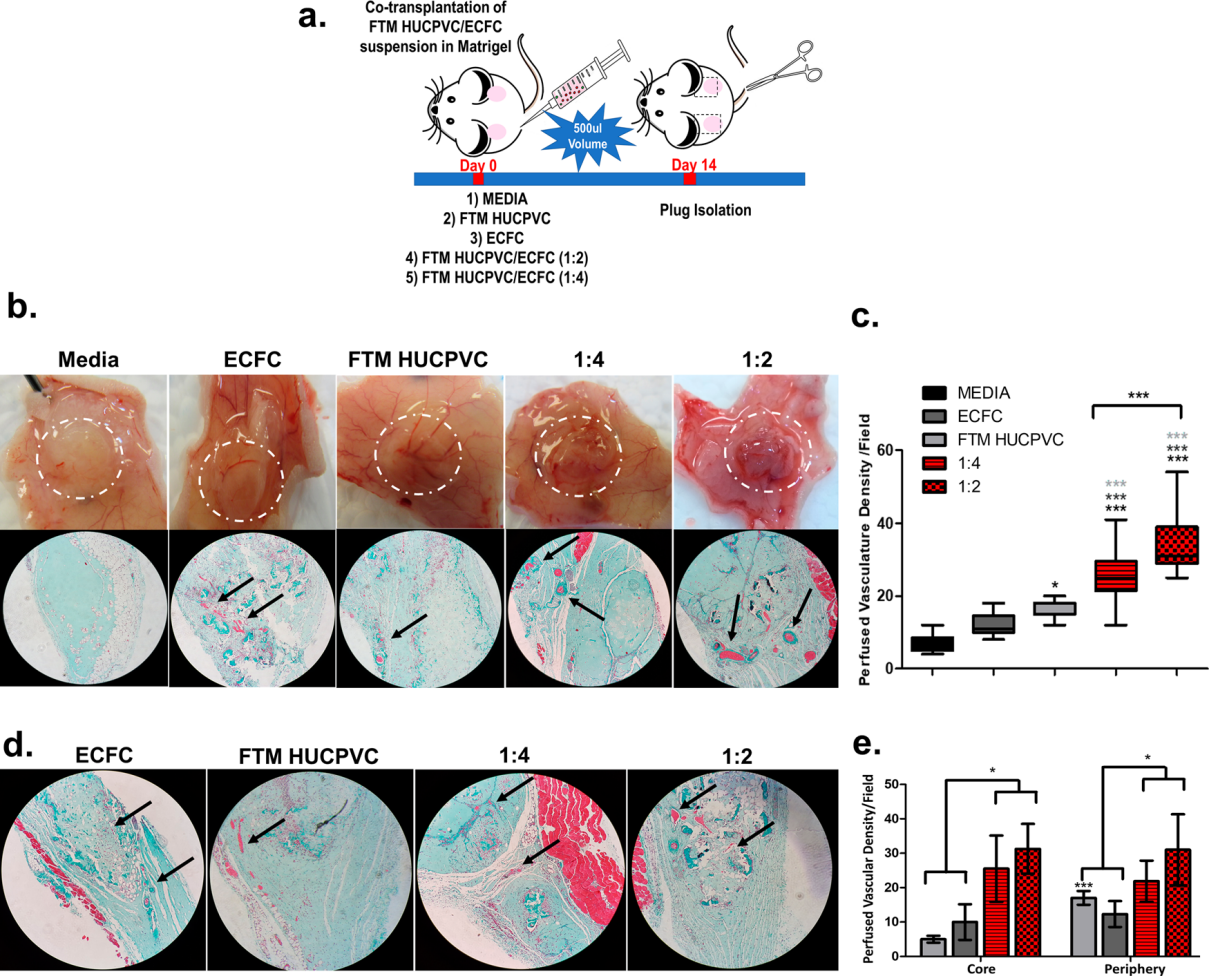
Co-injection of FTM HUCPVCs and ECFCs exhibit synergistic vascular potential compared to single injections in Matrigel plugs. **a** Matrigel plug study outline and treatment groups. **b** Bright-field images showing superficial vascularization of Matrigel plugs with combination and single FTM HUCPVC and ECFC treatments accompanied by Trichome staining of Matrigel plugs identifying perfused vasculature (black arrows). **c** Quantification of perfused vasculature within Matrigel plugs. *N* = 4 separate Matrigel plugs for each treatment group, *n* = 9–16 fields of sub-analysis. **d** Bight-field images showing the distribution (core vs. periphery) in Matrigel plugs (black arrows). **e** Sub-quantification of vasculature within the core and periphery of Matrigel plugs. *N* = 4 separate Matrigel plugs, *n* = 4–9 fields of sub-analysis. Statistical analysis by One-Way ANOVA using Tukey post-hoc test. *p < 0.05, *p < 0.01, ***p < 0.001. Bar graphs: data are presented as mean ± SEM.

FTM HUCPVCs and ECFCs were co-injected subcutaneously to quantify their potential for de novo vascularization in an in vivo nude mouse model (Fig. 7a). At 14 days, gross Matrigel plug perfusion was observed in all cell treatment groups, with increased perfusion in the combination groups (Fig. 7b). Masson’s trichrome staining identified significantly more perfused vasculature in the 1:2 combination treatment compared to 1:4 combination group *(p* < 0.001), single FTM HUCPVC and ECFC treatment groups *(p* < 0.001; Fig. 7c). Perfused vasculature was further quantified based on the location within Matrigel plugs (Fig. 7d). Combination treatments resulted in increased perfused vasculature at both the periphery and core of plugs compared to single cell treatments (*p* < 0.05).

### RNA-silencing of PDGFR-β in FTM HUCPVCs negatively impacts angiogenic potential in vitro and in vivo

**Fig. 8:**
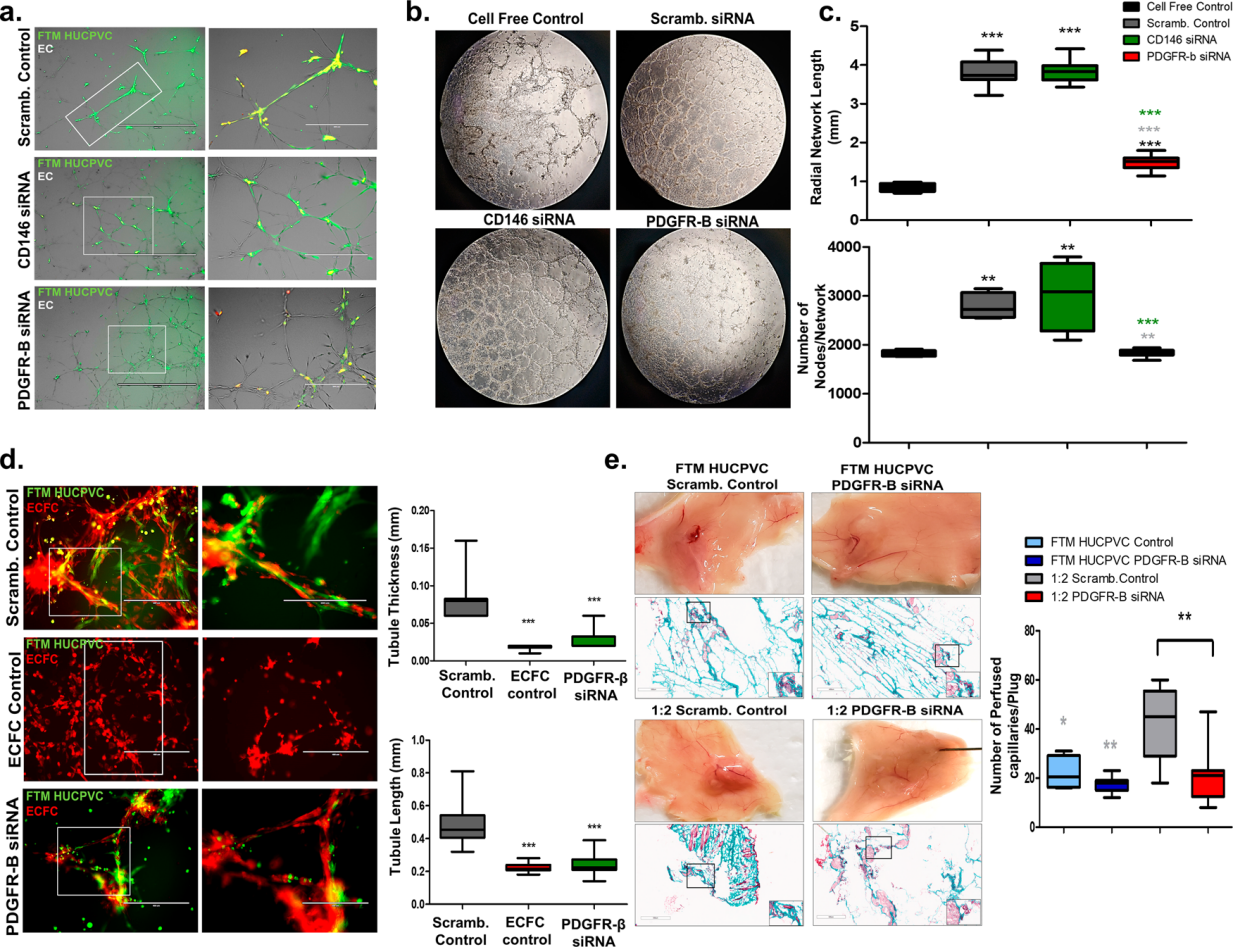
Silencing PDGFR-β expressed by FTM HUCPVCs impairs homing and angiogenic potential of FTM HUCPVCs with ECFCs. **a** Fluorescence imaging of transfected FTM HUCPVCs (green) co-cultured with aortic ring-derived endothelial networks (red) for 24 h. Low magnification scale bar: 1000 μm; high magnification scale bar: 400 μm. **b** Bright field images of aortic ring-derived endothelial networks co-cultured with PDGFR-β siRNA transfected FTM HUCPVCs (Day 3). **c** Quantification of network length and number of nodes in the aortic ring assay. Bright field images scale: ×10 magnification. *N* = 3 separate experiments with different donor lines, *n* = 4–6 fields of sub-analysis. **d** Fluorescence imaging of PDGFR-β siRNA transfected FTM HUCPVCs (green) with ECFC-derived vascular networks (red) at 24 h. Quantification of tubule thickness and length at 3-days following co-culture. (Gray asterisks represent significant differences between FTM HUCPVC and Scrambled (Scramb.) control ***p < 0.001). Low magnification scale bar: 400 μm; high magnification scale bar: 200 μm. *N* = 3 separate experiments with different donor lines, *n* = fields of sub-analysis. **e** Bright field images of Matrigel plugs paired with Masson’s Trichrome staining to identify perfused vasculature following co-injection of PDGFR-β siRNA transfected FTM HUCPVCs with ECFCs. Quantification of perfused vasculature within Matrigel plugs following cell injections. (Gray asterisks represent significant difference from 1:2 Scrambled control *p < 0.05, **p < 0.01) *N* = 4 separate Matrigel plugs for each treatment group, *n* = 4–9 fields of sub-analysis. Statistical analysis by One-Way ANOVA using Tukey post-hoc test for both assays. *p < 0.05, *p < 0.01, ***p < 0.001. Box plots: the black center line denotes the median value (50th percentile), while the black box contains the 25th to 75th percentiles of dataset. The black whiskers mark the 5th and 95th percentiles.

To investigate whether PDGFR-β expression by FTM HUCPVCs is necessary for mediating pro-angiogenic effects when combined with ECFCs, PDGFR-β was downregulated using siRNA transfection and a neutralizing antibody. In a separate treatment group, CD146, another constitutive, pericyte-associated marker was targeted by siRNA treatment. Both were compared to scrambled control (Supplementary Figs. 13–15). At 24-h post-culture, decreased homing, endothelial coverage, and network growth were observed following the downregulation of PDGFR-β but not CD146 when compared to scrambled controls in the aortic ring assay (ARA) (Fig. 8a, b). PDGFR-β downregulation and cell-free controls showed a significant decrease in network growth (*p* < 0.001) and network nodes (*p* < 0.01) compared to CD146 siRNA and scrambled siRNA transfected FTM HUCPVCs (Fig. 8c). FTM HUCPVCs with downregulated PDGFR-β showed decreased homing and integration within ECFC structures at day-3 of co-culture (Fig. 8d). Attenuation of PDGFR-β resulted in ECFC networks with significantly thinner (*p* < 0.001) and shorter tubules (*p* < 0.001), compared to scrambled siRNA transfected FTM HUCPVCs in ECFC co-cultures (Fig. 8d). To translate these effects in vivo, FTM HUCPVCs with attenuated PDGFR-β expression were co-injected with ECFCs in Matrigel plugs. FTM HUCPVCs with diminished PDGFR-β expression resulted in significantly fewer perfused capillaries in the 1:2 combination treatment compared to FTM HUCPVC scrambled control (*p* < 0.01; Fig. 8e). These mechanistic studies suggest that FTM HUCPVC-expressed PDGFR-β is required for cell homing and integration, necessary for in vitro and in vivo vascularization.

## Discussion

Favorable tissue remodeling particularly in the heart is multifaceted and requires a balance of multiple regenerative mechanisms that target specific stages of healing. Previous studies involving single-based therapeutics have proven to be more difficult than initially thought since a single cell source may not target all aspects of regeneration. The combination of potent trophic factor secretion including pro-survival signals are required to rescue revivable injured tissue and prevent further tissue loss. Pro-inflammatory responses are required to clear cellular debris to accommodate new tissue. This is followed by favorable ECM remodeling accompanied by angiogenesis, vasculogenesis, and pro-regenerative inflammation. The role of inflammation is important for different stages of regeneration but for the scope of this paper, immunocompromised rats were used to determine whether combination treatments improve cardiac function to greater degrees when compared to single-cell therapies and to characterize the pro-regenerative mechanisms specific to angiogenesis following ischemic injury.

This study integrates a systems biology strategy in combination with in vitro mechanistic studies to determine whether the co-transplantation of FTM HUCPVCs, a potent source of pericytes co-injected with ECFCs, a source of endothelial cells can improve cardiac recovery following ischemic cardiac injury and transition transient new blood vessels into functional vasculature. The co-transplantation of FTM HUCPVCs and ECFCs demonstrated synergistic improvements on cardiac vascularization, cardiac muscle preservation, and cardiac functional recovery, while limiting scar formation and apoptosis. Although single therapies of FTM HUCPVCs and ECFCs improved cardiac function compared to medium control, the combination of both cell types demonstrated additive effects. FTM HUCPVCs displayed potent paracrine and ECM remodeling properties, while ECFCs demonstrated potent vascular properties. Previous studies have demonstrated favorable regenerative properties of various MSC and ECFC sources individually, following ischemic injury^49,50^. More recently, studies involving the combination of two regenerative cell types highlighted a new avenue for cell-based therapies^36^. Here, we presented a small animal study of ischemic cardiac injury that specifically combined umbilical cord-derived MSCs with ECFCs and demonstrated significant improvements compared to single-cell therapy.

In vitro results highlighted the importance of direct cell-cell interactions between FTM HUCPVCs and ECFCs for favorable angiogenesis. Similarly, other sources of MSCs when co-cultured with endothelial progenitor cells (EPC), developed cell-cell contact for vascularization^51,52^. Transwell co-cultures with absent pericyte-endothelial direct interactions, displayed fewer and thinner tubules. Although ECFCs are vascular cells, the addition of FTM HUCPVC increased network growth and maturation, highlighting the role of pericytes for vascularization. FTM HUCPVCs displayed pericyte-like morphologies with extended cellular processes when co-cultured with ECFCs, while also expressing pericyte-associated markers, including PDGFR-β and CD146. The emergence of thicker tubules between networks, suggests tube maturation, which was observed only in the direct FTM HUCPVC-ECFC co-culture group compared to transwell cultures.

This present study demonstrated greater pericyte-like properties and angiogenic potential of FTM HUCPVCs compared to term HUCPVCs. Although term HUCPVCs demonstrated pericyte-like support of ECFC structures, significantly less coverage and network growth were observed compared to FTM HUCPVC-ECFC co-cultures. Protein analysis detected significantly lower levels of pro-angiogenic factors in term HUCPVC-ECFC co-cultures. Our study suggests greater pericyte and paracrine properties of FTM HUCPVCs compared to term HUCPVCs, translating to significantly greater vasculogenic effects in vitro. These differences highlight the importance of cell source, age, paracrine properties, and cell adhesion markers necessary for angiogenesis and therapeutic regeneration.

This study elucidated the key role of PDGF-β/PDGFR-β signaling in FTM HUCPVC-ECFC mediated vascular responses. Traditionally endothelial cells secrete PDGF- β as a chemotactic agent to recruit and dock pericytes via PDGFR-β^53,54^. FTM HUCPVCs expressed PDGFR-β, while ECFCs were a source of PDGF-β following co-culture (Supplementary Fig. 11). Liang et al. highlighted the importance of PDGF-β signaling in EPC and MSC co-cultures by utilizing neutralizing antibodies^51^. Similarly, silencing PDGF-β at multiple time points following MI, resulted in disorganized vessels and deleterious structural changes in the myocardium^55^. FTM HUCPVCs with impaired PDGFR-β expression had clustered morphologies, showed reduced homing, and impaired endothelial network growth in the ARA and ECFC co-cultures. Disrupting PDGFR-β/PDGF-β signaling decreased the overall number of perfused blood vessels in Matrigel implants.

Combination treatments showed a 10% improvement in cardiac function early as 5-days, which may be necessary for immediate cardioprotective effects to have incremental benefits over time. A recent meta-analysis showed an overall ~7.5% improvement in ejection fraction following MSC-based therapy^56^. Rodent models increased ejection fraction by 20% and 5–7% compared to control groups in large animal models of MI^57^. Despite similar reductions in scar size for combination groups and single FTM HUCPVC transplantation, the function of the scar and LV varied between both groups. The 1:2 combination improved ejection fraction (~139%) relative to medium control compared to FTM HUPVC single treatments (~16%), highlighting the potency of a combined FTM HUCPVC and ECFC cell therapy.

Apoptosis is a controversial mechanism in regeneration because it contributes to both beneficial and deleterious effects depending on the stage of wound healing^58,59^. Despite the duality effects of apoptosis, many researchers target apoptosis to prevent adverse cardiac remodeling and heart failure. A reduction in apoptosis was detected by cleaved caspase-3 signals in combination cell therapy treated LVs compared to single-cell treatments. Cardioprotection, observed as early as 5 days post-treatment, may be a contributing factor to the documented cardiac recovery at 4 weeks.

Gap junction proteins including Cx-43 play critical roles in mediating intra-myocardial communication for coordinated contractile function. Weakening and a decline in the number of gap junctions exacerbates cardiac fibrosis by activating fibroblasts^60^. Lentiviral-based overexpression of Cx-43 in the infarcted myocardium provided long-term protection against cardiac arrhythmias compared to control groups^61^. At 4-weeks, increased sarcomeric actinin and Cx-43 were detected in both combination treatments compared to groups. These results translated into a significant reduction in collagen scar formation in the 1:2 combination treatment group compared to single-cell groups at week 4.

Quantification of human FTM HUCPVC-secreted growth factors in LV identified ANG, ANGPT2, FGF-1, VEGF-C, and chemoattractants, including SDF-1 and GM-CSF, previously implicated in myocardial repair^62^. ANGPT1 is secreted to promote vascular stabilization while ANGPT2 is detected at sites of vascular remodeling and mural dissociation^63,64^. Several researchers reported increases in ANGPT2 at both RNA and protein levels following MI, whereas ANGPT1 levels decreased^65^. Higher ratios of ANGPT2:ANGPT1 were identified in the 1:2 combination treatment. Increased FTM HUCPVC-secreted ANGPT2 may enhance neovascularization in the ischemic myocardium. In this model, increased levels of VEGF-C were also detected in both FTM HUCPVC-only and 1:2 combination treatments. Studies have correlated increased levels of ANGPT2 with increases in VEGF levels following MI^65,66^. It was postulated that ANGPT2 along with VEGF are released at earlier stages of angiogenesis and vascular remodeling. This release removes the quiescent control of ANGPT1 on endothelial cells and induces vessel sprouting^65,67,68^. The presented results may further support the coordinated role of ANGPT2 and VEGF in vascular remodeling at earlier stages of cardiac repair.

Limitations of this study are primarily rooted in the model itself. The functional data from this study and parallel ongoing studies suggest that we have reached the maximum possible improvement that can be achieved in this model. Larger animal models including porcine models can accommodate up-to 15–20 cell injections in the LV, which may be more clinically feasible using a catheter instead of thoracotomy. Secondly, the age of animals does not represent patients who may receive this therapy in a clinical setting. Clinical data has demonstrated that aged patients have fewer and less functional circulating progenitor cells. Aged animal models may highlight age-associated impacts on cell therapy efficacy. Additionally, Foxn1^rnu^ rodents used in this study were immunocompromised, which prevents the evaluation of immunomodulatory properties of cell therapy and host immune responses to therapy. Studies involving immunocompetent animals are required to understand the interplay between allogeneic therapies and the host immune system. This is particularly important because human blood is neutrophilic, whereas rodents have abundant lymphocytes with variations in macrophage polarization^69,70^. Further analysis is required to characterize the interplay of the proposed combination treatment with the immune system for cardiac recovery. The presented study focused on regenerative mechanisms related to tissue remodeling, angiogenesis, and tissue preservation. By no means is this a complete framework of regeneration. Additional mechanisms including the recruitment of endogenous host immune cells, progenitor cells (CD34^+^, c-kit^+^), blood cells, and their effects on cardiomyogenesis, cell–cell communication, and proliferation are impportant^71^. Future work is necessary to identify and characterize the activation of host regenerative cells and the balance of pro-inflammatory and anti-inflammatory phenotypes for favorable cardiac microenvironments following ischemic injury.

Identification and characterization of favorable clinical outcomes specific for the mechanisms of disease are necessary. The CONCERT-HF trial showed benefit in reducing major adverse cardiac effects and improved the quality of life between combination cell therapy and placebo. Despite positive clinical outcomes, physiology surrogates including ventricular function or cardiac structure were not altered. This suggests that indirect measurements of physiological surrogates or biomarkers may not correlate with benefit-to-risk ratio. A combined approach investigating the patient walk test, quality of life, everyday function, and survival with physiological surrogates (endothelial cell function (nitric oxide response), vessel intima-media thickness, ventricular perfusion, and function is necessary. This is challenging to conduct in animal models but physiological surrogates are important for clinical trial development. If proposed therapies do not show improvements in physiological surrogates, it is less likely that these therapies would be tested in clinical trials. Identification of clear clinical outcomes for ischemic cardiomyopathies including mortality, functional/physical testing, risk of illness including stroke, and the quality of life combined with pertinent physiological surrogates including ventricular perfusion and function may be important. Lastly, variability in the regenerative capacity between FTM HUCPC samples is recognized. Pre-screening of cell lines using benchmark potency assays prior to both pre-clinical and clinical studies is required. For this study, ECFCs were isolated from multiple young animals, where cells were pooled from two donors. The method of pooling is not clinically applicable due to immunological risks. In vitro strategies are required to enhance the number and potency of isolated aged human ECFCs prior to injection, while limiting cell differentiation.

Despite ongoing investigations on the safety profiles of allogeneic sources, further studies are required to identify which regenerative cells are optimal for specific tissue injuries and microenvironments. Studies show that cardiac microenvironments due to free radicals are toxic to implanted cells in addition to diminished efficacy of autologous cells isolated from post-MI donors^72,73^. Ischemia-mediated activation of pro-inflammatory responses may impact the bone marrow niche and alteration of peripheral circulating cells^21^. This is an important factor to consider if using autologous sources of ECFCs, which are currently under investigation for acute MI in a placebo-controlled clinical trial^74^. In this present study, ECFCs with uniform expansion time, phenotypic profiles, age of animals, and the number of transplanted cells were consistent. This may not be feasible in the clinic, especially for autologous therapy due to varying patient age, extent of ischemic injury, and the number of harvested and expanded ECFCs. Since aged autologous sources of ECFCs pose a challenge, further investigations of priming or boosting autologous ECFCs with cytokines or transfection with eNOS are potential options. Since ECFCs demonstrate immunomodulatory and immunoprivileged properties, using allogeneic ECFCs from human cord blood or Wharton’s jelly may be clinically relevant. Studies show that cord blood-derived ECFCs are tolerated and promote angiogenesis in an immunocompetent mouse model^75^. While cord blood and umbilical cord banking has become more popular and affordable, it is promising that future patients may use autologous, young, potent therapeutic cells for healing injured tissue.

This study describes the assessment of a new a source of MSC, derived from the first-trimester umbilical cord, for the purpose of regenerative angiogenesis. FTM HUCPVCs are an accessible source of young MSCs, where the combined delivery of FTM HUCPVCs and ECFC demonstrated synergistic effects on tissue vascularization, reducing apoptosis, favorable ECM remodeling, and promoting cardiac functional recovery. The combination of both cell types improved multiple regenerative aspects of tissue remodeling following MI. These results further support the hypothesis that a single source of therapeutic cells may not efficiently target all regenerative mechanisms in the heart following MI. The application of combined cell-based therapies including cells with unique regenerative but synergistic properties can collectively improve cardiac recovery following ischemic injury by targeting multiple regenerative mechanisms.

## Methods

### Ethics approval and umbilical cord collection

The study, including the collection of first-trimester human umbilical cords (8–10 weeks of gestation) from elective pregnancy termination and full-term human umbilical cords, was approved by the University of Toronto Research Ethics Board (REB #28889), and complied with ethical regulations including the Declaration of Helsinki and Tri-Council Policy Statement: Ethical Conduct for Research Involving Humans (TCPS 2 (2022)). Term newborn cords were collected through a third-party source (Lifeline Stem Cell). Written informed consent was obtained from all human participants.

### Cell culture and phenotypic characterization

MSC lines from human first-trimester and term umbilical cords were previously established and characterized^19^. Cryopreserved HUCPVC (FTM and term HUCPVC) stored in liquid nitrogen were thawed at a constant temperature of 37 °C for 5 min^19^. Thawed cell suspensions (1,000,000 cells/ml) were 10x diluted in alpha-modified minimum essential medium eagle (α-MEM) (Gibco), supplemented with 5% human platelet lysate (HPL) (Compass Biomedical) and 1% penicillin/streptomycin (P/S) (Gibco). Cell suspensions were centrifuged at 1600 RPM for 10 min. Cell pellets were resuspended in 10 ml pre-warmed complete culture medium (a-MEM, 5% HPL, 1% P/S) and plated onto culture vessels. Cells were typically seeded at 2.5 × 10^3^ cells/cm^2^ in 10-cm^2^ culture dishes (Corning) and adjusted to the same ratios for different surface areas. Plated cells were incubated at 37 °C, 5% CO_2,_ and imaged daily to document cell morphologies and cell densities. HUCPVCs were passaged at 70–80% confluency using TrypleE (Life Technologies) dissociation solution (1 ml/2.5 cm^2^) for 5 min at 37 °C in 5% CO_2._ Dissociated cells were diluted 1:1 with complete culture medium and centrifuged at 1600 RPM for 10 min. Cell pellets were resuspended in 1 ml complete culture medium and HUCPVC were counted using Trypan blue (Life Technologies) staining, in an automated cell counter (Invitrogen). The cells were either plated again for further culture expansion or processed for experimental assays. HUCPVCs at passage 6–7 were utilized for in vitro and in vivo experiments. Experiments were replicated with different cell donors, n values listed in figures.

ECFCs were isolated from bone marrow aspirates from 8-week-old Sprague–Dawley male rats as described previously^76^. All animal procedures were conducted and reported according to ARRIVE guidelines and approved by the Animal Care Committee of the University Health Network (Toronto, Canada). All studies were performed with institutional research ethics board approval (AUP 4276, University of Toronto, Toronto, Canada). Bone marrow aspirates were collected from Sprague–Dawley male rats (8 weeks of age). Animals were euthanized in carbon dioxide chambers set to 20% gas replacement (flow rate = chamber volume × 0.2 per min). Hind limbs were removed by dislocating hip joints and placed in Hank’s balanced salt solution (HBSS) (Thermofisher) supplemented with 1% P/S. Fur and muscle were carefully removed, exposing the femur, tibia and fibula. Only the femur and tibia were utilized for bone marrow extraction. Epiphysis of bones were cut with surgical cutters and bone marrow cavities were flushed with HBSS (1%P/S). Flushed bone marrow cavities were filtered using 70μm filtering mesh (Corning). Diluted bone marrow aspirates were centrifuged at 1600 RPM for 10 min. Cell pellets were reconstituted with EGM (Lonza-same from chapter 2). Cells were seeded onto T75 (Sarstedt) flasks with 15 ml of EGM and incubated at 37 °C in 5% CO_2_. Following 48 hours, medium was removed. Flasks were rinsed with PBS (x2) with strong tapping to remove all non-adherent cells. Cells were again cultured with 15 ml of fresh EGM and culture medium was changed every three days. Cell colonies were imaged regularly, where multiple ECFC colonies were observed at day 14 of culture. Depending on the success of bone marrow isolations, ECFC cultures were ready for experiments in 14 ± 2 days. For in vitro experiments, ECFCs were pre-labeled with Cell Tracker Orange™ (CTO) (Life Technologies) prior to cell detachment. 1 ul/ml of CTO was added to ECFC cultures for 30 min at 37 °C in 5% CO_2._ Following incubation, culture medium was removed and 8 ml of TrypleE was added. Culture flasks were incubated for 15 min with intermittent tapping to detach ECFC colonies. Detached cell suspensions were diluted (1:1) with EGM and centrifuged for 15 min at 1600 RPM. Cell pellets were reconstituted with EGM and cells were counted using Trypan Blue staining and automated cell counter (Invitrogen). ECFCs were never frozen and used fresh for all experiments.

Both cell types were characterized using flow cytometry prior to experiments (Supplementary Figs. 5–8).

### Phenotypic characterization of HUCPVCs by flow cytometry

For flow cytometry (FC) and fluorescence associated-cell sorting (FACS), cell cultures were dissociated and counted as mentioned above. Cell suspensions were incubated with fluorophore-conjugated primary antibodies, according to the provider’s description. For most antibodies, 50,000 cells were incubated with 2.5 μl of antibody for 20 min at 4 °C. FC analysis was performed using either a MACQuant analyzer (Miltenyi Biotec; Create Fertility Centre, Toronto) or digital (LSR II, Canto II, BD; UHN SickKids Flow Cytometry Facility, Toronto) analytical cytometers. FACS was performed using digital cell sorters (MoFlo Astrios, Aria II, UHN SickKids Flow Cytometry Facility, Toronto). Human-specific TRA-1-85^+^ sorted cells were collected in lysis buffer (Qiagen) for qPCR and next-generation sequencing (NGS). Fluorescence signals were gated based on unstained populations and gates were set at 10^1^ fluorescence intensity decades. Mean fluorescence intensities were used for analysis. Gating strategies included in figures. Antibodies used for identifying HUCPVC phenotypes included CD90-APC (#130-114-903), CD105-APC (#130-098-778), CD146-FITC (#130-111-323), HLA-G-FITC (130-112-004) at a 1:40 dilution (Miltenyi), and PDGB-R-APC (R&D # FAB1263A) at 1:20 dilution (Supplementary Table 1).

### Phenotypic characterization of ECFCs by flow cytometry

For flow cytometry (FC) and fluorescence-associated cell sorting (FACS), cell cultures were dissociated and counted (mentioned above). Cell suspensions were incubated with fluorophore conjugated primary antibodies according to the providers description. For all antibodies, 50,000 cells were incubated with 2.5 μl of antibody for 20 min at 4 °C. FC analysis was performed using the MACQuant analyzer (Miltenyi Biotec; Create Fertility Centre, Toronto). FACS was performed using digital cell sorters (MoFlo Astrios, Aria II, UHN SickKids Flow Cytometry Facility, Toronto). Fluorescence signals were gated based on unstained populations. Gates were set at 10^1^ fluorescence intensity decades. Antibodies for ECFC FC were as follows: CD31 (AF3628 R&D-FITC), CD34 (AF6518 R&D-FITC), CD133 (NB120-16518 Novus Biologicals-PE), CD38 (50-0380-80 Thermofisher-APC), KDR (NB200-208 Novus Biologicals-PE), CD146 (560846 BD sciences-FITC), and VE-Cadherin (BS-0878R-A488 BioUSA-FITC) and CD117 (20-1172-u025- Tonbo Science-APC). All antibody titrations were performed and used at a concentration of 1:20 (Supplementary Table 2).

### Model of myocardial infarction and cell transplantation

All animal procedures were approved by the Animal Care Committee of the University Health Network, following ARRIVE guidelines (AUP#4276.6, 893.35, 1133.21) (Toronto, Canada). NIH-*Foxn1*^*rnu*^ rats (Charles River) underwent left anterior descending coronary artery (LAD) ligation to induce MI. Eight-week-old, male nude rats (NIH-*Foxn1*^*rnu*^*-Charles River)* were anesthetized (2% isoflurane), analgised (0.05 mg/kg buprenorphine), intubated, ventilated (2% isoflurane), and placed on a heated mat. Left-side thoracotomy was performed between the 4^th^ and 5^th^ ribs, the pericardium was opened, and the left coronary artery permanently ligated 2–3 mm from its origin with a 7–0 polysuture. White discolouration of the myocardium distal to the ligature confirmed successful infarction. Cyclosporine (5 mg/kg) and buprenorphine (0.05 mg/kg) were administered post-operatively for five days. At 5 days post-MI, cardiac function of all rats was evaluated using echocardiography and those with fractional shortening of 20–40% were randomly separated into 6 further groups (*n* = 9–12/group): **G1**: Medium injection; **G2**: undifferentiated FTM HUPVCs (3 × 10^6^ cells/rat); **G3**: undifferentiated FTM HUPVCs (1 × 10^6^ cells/rat); **G4**: FTM HUPVCs (6 × 10^5^ cells/rat) **G5**: ECFC (2.2 × 10^6^ cells/rat); **G6**: FTM HUCPVC and ECFC (1:2) (total cells count 3 × 10^6^ cells/rat); and **G7**: FTM HUCPVC and ECFC (1:4) (total cell count 3 × 10^6^ cells/rat). ECFC single injections included the average number of ECFCs that were injected in the 1:2 and 1:4 treatment groups (2 × 10^6^ and 2.4 × 10^6^ respectively). A total volume of 50 µl of cell suspension or equivalent volume of medium alone was injected into 3 peri-infarct areas, where animals were kept under anesthesia (2% isoflurane). Personnel injecting treatments were blinded to treatment groups. All groups had end-point analyses at 5 days and 4 weeks post cell injections. ECFC for these studies were obtained from bone marrow of 8-week-old Sprague–Dawley (SD) rats. ECFC and FTM HUCPVC were pre-labeled with Qtracker (Q-dot) (Thermofisher) dye prior to myocardial injection for the 5-day study.

### Echocardiography

Cardiac function was measured by echocardiography before MI, day of cell injection (Day 0), 5 days, 2- and 4-weeks following cell injection. Rats were anesthetized as described above and echocardiography was undertaken with a GE Vivid 7 ultrasound system (GE Healthcare Canada) with a 10 S transducer (frequency 11.5 MHz, depth *2* cm). Short-axis views were obtained from the parasternal approach. Blinded left ventricle (LV) dimensions (left ventricular end-diastolic internal diameter (LVIDd) and end-systolic internal diameter (LVIDs) were measured in M-mode. Ejection fraction was calculated as (LVIDd^3^ - LVIDs^3^) / LVIDd^3^ × 100. Fractional shortening was calculated as (LVIDd - LVIDs) / LVIDd × 100.

### Pressure–volume catheterization

Cardiac function was measured by a pressure-volume catheter as an end point assessment at 4 weeks post cell treatments. Animals were anesthetized (2% isoflurane), left-side thoracotomy was performed between the 4th and 5th ribs, the pericardium was opened, and sternotomy was performed. A 2.0 F micromanometer catheter (SPR8-838 Millar Instruments, Houston TX) was inserted through the myocardium into the left ventricle of anaesthetized animals. Pressure-volume changes were continuously sampled (1000/s) using a pressure-conductance system connected to a PowerLab/4SP analog to digital converter (AD instruments). Resulting pressure-volume loops were used to assess cardiac function (ejection fraction, end-systolic and diastolic volumes, dP/dt and tau) with PVAN 3.3 (Millar Instruments) software. Following this procedure, the aorta was cannulated with a 4 ml vacutainer containing EDTA (367861, BD) and blood collected for subsequent analysis. Hearts were arrested in diastole with 1 ml of 10% KCl and snap frozen in liquid nitrogen or fixed in 2% PFA for biochemical analysis or cryo-sectioning respectively.

### Cell retention studies

Five days after cell treatments, hearts were collected, the left ventricle (LV) was excised and snap frozen in liquid nitrogen. For cell retention studies, human DNA content measurement was calibrated against human genomic DNA in rat hearts injected with 600k, 1 million, and 2 million FTM HUCPVC, where left ventricles were isolated within 30 min following injections (Day 0). Cell retention percentages were determined based on relative dose injected at time zero. Alu primers (IDT) were designed and qPCR was used to measure human cDNA in the LV. Ct values less than 28 were excluded.

### Histological analysis

Fixed hearts were sectioned beneath the ligature into four 2 mm sections and prepared for cryo-sectioning through 10, 20, and 30% sucrose suspension. Frozen sections were processed by histological staining and immunohistochemistry. Scar size was determined from Masson’s trichrome stains and expressed as a percent of total left ventricle area.

### Immunohistochemistry

Sections were washed (2 × 5 min) in tris-buffered saline (TBS) (Thermofisher) containing 0.025% TritonX (Millipore) and blocked in TBS containing 10% normal goat serum (Thermofisher) and 1% BSA for 1 h. Primary antibodies were diluted in TBS (1% BSA) and applied overnight at 4 °C, secondary antibodies were diluted at 1:500 and applied at room temperature for 1 h. Anti-Connexin 43 (Abcam-ab11370), anti-sarcomeric actinin (Abcam-ab32575), anti-PDGFR-β (Abcam-ab62437); Isolectin GS-IB4 (Life technologies-l-21412) and BODIPY Thapsigargin (Life technologies-B-7487); cleaved caspase-3 (Cell Signaling Technologies-9661S). All primary antibodies were used at 1:200 dilution except for IB4 (1:50). (Supplementary Table 3). Respective secondary conjugated antibodies were purchased from Thermofisher and used at a 1:500 dilution (Supplementary Table 4). Samples were coded; therefore, imaging and analysis was performed blinded. Image analysis was conducted using Fiji software (Image J)^77^.

### In-situ zymography

DQ Gelatin (Life Technologies) was applied to assess matrix metalloproteinase activity. Sections were renatured with PBS containing 2.5 % triton for 15 min and assay was performed according to the manufacturer’s instructions.

### Multiplex luminex elisa assay

Custom Luminex Multiplex Elisa was designed using R&D systems custom tool, targeting 11 human-specific analytes. 200 mg of left ventricle homogenates were further lysed with proteinase inhibitors and 200 µl of PBS (*N* = 3). Ventricle homogenates were centrifuged at 1600 RPM for 2 min. Supernatants were collected and protein was quantified using Qubit protein assay kit (Thermofisher). Approximately 1.25 mg total protein was processed using manufacturer protocol (R&D). Fluorescent beads (PE) tagged with proteins were processed using flow cytometry (MACQuant analyzer, Miltenyi Biotec) to detect PE signal. Instrument settings were determined by processing control beads untagged with protein. Data analysis was conducted using FlowJo software. Mean fluorescence intensities were normalized to number of beads detected per sample.

### Tissue-level cytokine analysis (Rat Proteome Profiler)

In all, 50 mg of left ventricle homogenates were further homogenized in 500 μl PBS and protein inhibitors. Protein was quantified using Qubit protein assay kit (Thermofisher) and ~2.4 mg total protein was processed using Proteome Profiler Rat XL Cytokine Array kit (R&D). Protein arrays were analyzed using the HLImage + + software. Pixel densities were normalized to internal positive controls (100%) and expressed as percentages of positive control.

### Matrigel plug assay

Eight-week-old NU*-Foxn1*^*rnu*^ mice were injected subcutaneously with Matrigel combined with either 250 μl FTM HUCPVC-single injection (4 × 10^3^ cells/ul), ECFC-single injection (4 × 10^3^ cells/ul), 1:2 or 1:4 combination ratios (4 × 10^3^ cells/ul total) or cell-free medium injections). Following 14-days, Matrigel plugs were isolated and fixed in formalin for vasculature quantification. For transfection studies, FTM HUCPVCs were transfected with 25 ρM validated small interfering (si) RNAs targeting PDGFR-β, CD146, and control siRNAs using lipofectamine RNAiMAX Transfection Reagent and co-injected with ECFCs.

FTM, TERM HUCPVC, and ECFC were cultured using the methods mentioned above. Treatment groups included FTM:ECFC combinations (1:2, 1:4) using two independent FTM HUCPVC lines, term HUCPVC, HUCPVC, ECFC, and cell-free medium treatments. A total of 1,000,000 cells were prepared for each Matrigel plug injection in a volume of 1000 ml. The ratio of FTM HUCPVC and ECFC were adjusted to a total of 1,000,000. Prior to injections, both cell types were mixed and placed on ice. Prior to injections, 500 µl of cell suspensions were mixed quickly with 500 μl of Matrigel on ice. Matrigel mixed-cell suspensions were injected subcutaneously in FoxN1^nu^ 8-week-old male mice using a 22.1/4-gauge needle. Injected plugs were held with forceps until the Matrigel plugs polymerized. Each mouse was injected to carry 4 Matrigel plugs, containing a plug from each different cell treatment group (*N* = 4). Matrigel plugs were retrieved 14-days following injection. Dorsal skin was excised along the vertebral column and carefully opened to visualize Matrigel plugs. Macroscopic images were taken to observe superficial vascularity and blood coagulation within Matrigel plugs. Matrigel plugs were fixed in formalin for 48 hours, embedded in paraffin, and sectioned (5 micron). Sectioned tissue underwent Masson’s Trichrome staining to visualize perfused vasculature and changes in the Matrigel matrix. Blood vessel density was quantified using ImageJ software and stratified based on regions within the Matrigel plugs.

### ECFC tube formation assay

Twelve-well plates were pre-coated with 300 μl of Matrigel (phenol red with growth factors) and incubated for 30 min (37 °C, 5% CO_2_). Polymerization was attenuated with 200 μl of EBM. HUCPVC and ECFC were pre-stained with Cell Tracker™ Green and Orange respectively for 20 min at 37 °C, 5% CO. Pre-stained cells were washed and resuspended in EGM. HUCPVC and ECFC cell suspensions were mixed at 1:2 (33k:66k) or 1:4 ratio (20k:80k) ratios for a total of 100,000 cells/12-well respectively. Mixed cell suspensions were added to Matrigel-coated wells and topped with 1 ml EGM. For transwell (TW) co-cultures, ECFC were seeded onto Matrigel-coated wells while HUCPVC were seeded on TW inserts (0.4um). TW inserts were placed into ECFC-seeded wells and 500 μl of EGM were placed on top and below TW inserts. Conditioned media was analyzed for human-derived proteins. Cells were isolated and processed for next-generation sequencing using the Ion PGM sequencer as described below.

### Imaging and quantification of endothelial networks

For HUCPVC-ECFC co-cultures, bright field (Olympus) microscopy was used to visualize initiation of endothelial structures. At least 4 phase-contrast images were taken to capture ECFC-derived networks. Tubular lengths and thickness were documented for up to 14 days. Quantification of network properties was performed using ImageJ software, utilizing the angiogenesis plugin on day 3 co-cultures. Fluorescence microscopy (EVOS™; LifeTechnologies) was used to visualize physical interactions between HUCPVC and ECFC. Fluorescence images allowed for tracking HUCPVC in relation to ECFC-derived vascular structures.

### Human and rat-specific proteome profilers

Conditioned medium from HUCPVC and ECFC co-cultures were collected at different time points throughout assay setup (24 h, 48 h). To determine changes in HUCPVC-secreted proteins following co-culture, the Proteome Profiler™ Human Angiogenesis Array Kit (ARY007, R&D) was used. Conditioned medium was collected, centrifuged and the supernatant was used for arrays. Proteinase inhibitor cocktail (Thermofisher) was added to supernatants and samples were processed according to manufacturer’s protocol. To detect changes in rat endothelial cell secreted proteins following co-culture, the Proteome Profiler Rat XL Cytokine Array Kit (ARY030) was used. Conditioned medium samples were processed as above, and samples were processed according to manufacturer’s protocol. Protein arrays were analyzed using the HLImage + + software. Pixel densities were normalized to internal positive controls (100%) and expressed as a percentage of positive control.

### Processing endothelial structures for downstream analysis

Conditioned medium was collected at 24 hours following co-culture in tube formation assays. Conditioned medium was centrifuged at 1600 RPM for 5 min, supernatant collected, and stored at -80 °C for subsequent protein analysis. At assay endpoint, endothelial networks were washed with phosphate-buffered saline (PBS) (Sigma-Aldrich) and disrupted to harvest cells from Matrigel. Briefly, 300 μl of Dispase™ (Stemcell Technologies) were added to each well and incubated at 37 °C, 5% CO for 15 min. Following incubation, quick aspirations and release were conducted to disrupt Matrigel-embedded endothelial networks. Cell suspensions including Dispase and digested Matrigel were collected in 15 ml falcon tubes and this process was repeated (3x). The viability of harvested cells ranged between 55–70% and, approximately 80–100 K cells were harvested. Cell suspensions were centrifuged at 1600 RPM for 6 min. Following the removal of supernatant, cell pellets were resuspended in 3 ml of trypsin and incubated for 10 min (37 °C, 5% CO). EBM was added (1:1) to neutralize trypsin. Cell suspensions were centrifuged at 1600 RPM for 6 min. Supernatants were carefully removed, and cell pellets were either resuspended in PBS supplemented with 3%FBS for FC/FACS or resuspended in lysis buffer (Qiagen) for qPCR/NGS.

### Aortic ring assay set-up

The aortic ring assay was established as done previously^26^. All animal procedures were conducted and reported according to animal research reporting of in vivo experiments (ARRIVE) guidelines and approved by the Animal Care Committee of the University Health Network (Toronto, Canada), (AUP 4276, University of Toronto, Toronto, Canada). Aortic tissues were isolated from Sprague–Dawley female rats of reproductive age (week 9). Animals were euthanized in carbon dioxide chambers set to 20% gas replacement (flow rate = chamber volume × 0.2 per min). Aortas were exposed by an excision through the chest cavity and lung tissue was removed. Aorta’s were identifiable adjacent to the vertebral column. Using surgical tools, the thoracic aorta was excised and cut into ~1 mm sections, yielding approximately 15–20 rings. Matrigel™-phenol red with growth factors (200 μl) (Corning) was coated evenly on 12-well plates (on ice) and then placed in a humidified incubator (37 °C, 5% CO_2_) for 30 min. Once the Matrigel was polymerized; a freshly obtained aortic ring was placed at the center of each well. Then 300 μl of Matrigel was carefully applied on top of the aortic ring tissue and incubated for 30 min (37 °C, 5% CO_2_). Once polymerized, 1000 μl of pre-warmed endothelial growth medium supplemented with FGF, VEGF, EGF, IGF, hydrocortisone, ascorbic acid, heparin and gentamicin (Endothelial complete medium- EGM, Lonza) were added. EGM was removed 24 hours following incubation and replaced with 1000 μl of endothelial basal medium supplemented with 2% fetal bovine serum (FBS) (Hyclone) and 1% P/S (EBM) for the remainder of the assay and replaced every 2 days. Bright field microscopy was used to monitor endothelial sprouts until endogenous endothelial networks (5–7 days) were ready for co-culture experiments. For direct co-cultures, HUCPVCs were harvested and prepared as single-cell suspensions using TrypLE (Invitrogen) at 37 °C for 5 min. HUCPVC were pre-stained with cell tracker green dye (CTG) (CellTrackerGreen™; Life Technologies) for 30 min, washed (1x) and resuspended in EBM + 2% FBS, 1% P/S. Using an automated cell counter, approximately 10,000 pre-stained HUCPVC were added to the aortic ring assay endothelial networks (Day 0).

### Imaging and quantification of endothelial networks

For the aortic ring assay, bright-field (Olympus) microscopy was used to document changes in network growth and structures. Phase-contrast images of four fields (4 quadrants of viewing field) were taken to measure radial network growth and total number of network closed loops up to.

7 days after addition of pre-labeled HUCPVC. Quantification of network growth and network loops was performed using ImageJ software, utilizing the angiogenesis plugin, on day 5 of co-culture. Fluorescence microscopy images (EVOS™; Life Technologies) were taken to evaluate HUCPVC-mediated ECM processing and migratory potential. Fluorescence images could identify preferential homing of HUCPVC to different regions of aortic networks and development of physical interactions between HUCPVC and endothelial cells during co-culture. Investigators were blinded to treatment groups.

### Targeted RNA sequencing of human HUCPVC from ECFC co-cultures using next-generation sequencing

RNA was isolated from HUCPVC/endothelial co-cultures (*n* = 3), HUCPVC alone cultures (*n* = 3) and ECFC single cultures using the Qiagen RNAeasy kit, according to the manufacturer’s instructions. RNA quantification and quality assessments were performed using the Qubit Fluorometer (ThermoFisher) and Bioanalyzer 2100 (Agilent Genomics) at the Princess Margaret Genomic Centre (Toronto, Canada). RNAseq libraries were prepared with Ion Ampliseq RNA library kit 2.0 (Invitrogen, Carlsbad, California, USA). Briefly, 10 ng of RNA was reverse transcribed using SuperScript IV VILO (Invitrogen Life Technologies, Carlsbad, California, USA) into cDNA and selectively amplified with a targeted RNA sequencing custom panel that included 108 amplicons as outlined in (Supplementary Table 5), according to the manufacturer’s instructions (Invitrogen). Primer sequences were partially digested, and barcode-adaptors (Ion Xpress Barcode Adaptors) were ligated to the amplified cDNA followed by purification using AMPure XP beads (Beckman Coulter) and additional amplification steps. Purified, amplified libraries were equalized to 23 pM using Ion Library TaqMan™ Quantitation Kit (ThermoFisher). Ion 318™ Chip Kit v2 chips (Invitrogen Life Technologies) were used for sequencing. Up to 24 samples were loaded per chip using 23 pM of the pooled libraries. NGS was performed using the Ion PGM sequencer, 400 flows (Invitrogen Life Technologies). Unaligned read files (FASTQ) were uploaded to Partek Flow (Partek) for bioinformatic analysis. Samples were trimmed for quantity, aligned to the rat genome (rn5) using Spliced Transcripts Alignment to a Reference (STAR) (v2.5.2)^78^, and subjected to post-alignment quality control. All unaligned reads were realigned to the human genome (hg19) using STAR (v2.5.2). Aligned files were subjected to post-alignment QC to determine the number of useable reads for each sample, percent alignment, and Phred quality score. Samples with greater than 100,000 total reads, showing greater than 98% alignment, were included in the analysis (and no samples were excluded based on this criteria). Targeted RNASeq analysis was performed by quantifying aligned reads to transcriptome using the Partek/EM platform and Homo sapiens (human) – hg19 canonical Transcripts as the genome build and the custom amplicon panel as the annotation model. Quantification was performed with strict paired-end compatibility and minimal read overlap, with the feature option set at 75% of read length. Transcripts were filtered (excluded if the maximum <5.0) and normalized using trimmed mean of M-values (TMM). Principal component analysis (PCA) was performed to assess the variability of the dataset. Differential gene expression and statistical analysis were performed by comparing each of the untreated HUCPVC to co-cultured HUCPVC. Transcripts were defined as differentially expressed if the fold change (FC) -2 > FC > 2 and False Discovery Rate (FDR) < 0.05.

### PDGFR-β silencing

siRNA for PGDFR-B (2 validated constructs s10242, s10241), CD146 (1 validated construct s8572), and scrambled negative control (4390843). All purchased from Thermofisher. Constructs were first diluted by 10 and final concentration used was 25ρmol as suggested by manufacture. FTM HUCPVC (25,000 cells) were plated in each well of a 6-well plate. Cells were cultured in basal α-MEM medium with 2.5% HPL (Compass Biomedical). Following 24 hours, alpha MEM was replaced with Opti-MEM factor-reduced medium (Life Technologies) supplemented with 2.5% HPL. Mixtures of siRNA with lipofectamine (Thermofisher) were administered to cells. Block-it^TM^ (ThermoFisher) fluorophore-labeled control RNA was used to visualize transfection efficiency. Conditioned medium was collected, and cells of different incubation groups were harvested every 24 h for protein and gene analysis (for 5 days). FTM HUCPVC were processed for flow cytometry and qPCR analysis to confirm successful downregulation of the target protein and its mRNA. To test other methods of silencing PDGFR-β, FTM HUCPVC were first pre-stained with Cell Tracker Green (described previously). Pre-stained FTM HUCPVC were processed similarly to that used for flow cytometry protocols. Approximately 50,000 FTM HUCPVC were incubated with FITC-conjugated, human-specific primary monoclonal mouse IgG antibody for PDGFR-β (2.5ug/100ul) (R&D AB-20-NA) and associated FITC isotype control (BD Biosciences 555748). for 20 min. Following incubation, FTM HUCPVC were washed and approximately 10,000 FTM HUCPVC were co-seeded in the aortic ring assay.

### Statistical analysis

Data are expressed as the mean ± standard error of the mean. Inter-group comparisons were performed using analysis of variance. If the analysis of variance F ratio was significant, differences were specified by Tukey post-hoc tests. All statistical analyses were performed using GraphPad Prism 6 (GraphPad). Differences of *p* < 0.05 were considered statistically significant. Data are displayed as box and whisker plots except proteome array results due to grouped analyses. Description of the box whisker plots: The center line, median; box limits, upper and lower quartiles; whiskers, 1.5x interquartile range; points and outliers. For groups without a whisker suggest either that the lower quartile is equal to the minimum or the higher quartile is equal to the maximum. Next-generation sequencing statistical analysis was performed using Partek Flow (Partek). Transcripts were defined as differentially expressed if the fold change (FC) -2 > FC > 2 and False Discovery Rate (FDR) < 0.05. *N* values are documented in figure legends for all experiments.

### Reporting summary

Further information on research design is available in the Nature Research Reporting Summary linked to this article.

### Supplementary information

Supplementary Files

Reporting Summary

## Data Availability

The datasets generated during and/or analyzed during the current study are available from the corresponding author on reasonable request. Sequencing data have been deposited in *Genome Sequence Archive (Genomics, Proteomics & Bioinformatics 2021) in National Genomics Data Center (Nucleic Acids Res 2022), China National Center for Bioinformation/Beijing Institute of Genomics, Chinese Academy of Sciences (GSA-Human: HRA004811) that are publicly accessible at* https://ngdc.cncb.ac.cn/gsa-human .Further information and requests for resources and reagents should be directed to corresponding author (Andrée Gauthier-Fisher, andree@createivf.com) or principal investigator (Clifford Librach, drlibrach@createivf.com).
