# Supplementary material for: Combination human umbilical cord perivascular and endothelial colony forming cell therapy for ischemic cardiac injury
Source: NPJ Regen Med. 2023 Aug 25;8:45. doi: 10.1038/s41536-023-00321-3 (PMC10457300; doi:10.1038/s41536-023-00321-3)
Supplement: Supplementary file 1 — Supplementary Files [file 41536_2023_321_MOESM1_ESM.pdf]

SUPPLEMENTARY TABLES AND FIGURES

Combination Human Umbilical Cord Perivascular and Endothelial Colony Forming Cell Therapy for Ischemic Cardiac Injury

Farwah Iqbal[1,2]*, Alexander Johnston[1], Brandon A. Wyse[1], Razieh Rabani[1], Poonam Mander[1], Banafshe Hoseini[1], Jun Wu[6], Ren-Ke Li[6], Andree Gauthier-Fisher*[1], Peter Szaraz[1] and Clifford Librach*[1,3,4,5]

Affiliations:

[1] Create Fertility Centre, Toronto, ON, Canada

[2] Virginia Tech Carillion School of Medicine, Roanoke Virginia, USA

[3] Department of Obstetrics and Gynecology, University of Toronto, Toronto, ON Canada

[4] Institute of Medical Sciences, Department of Physiology, University of Toronto, Toronto, ON, Canada

[5] Department of Obstetrics and Gynecology, Women's College Hospital, Toronto, ON, Canada

[6] Toronto General Research Institute (TGRI), University Health Network (UHN), Toronto, ON, Canada

*Corresponding author

Dr. Clifford Librach

Andree Gauthier-Fisher

790 Bay Street, Suite 420

Create Fertility Centre

Toronto, Ontario, M5G 1N8

drlibrach@createivf.com

andree@createivf.com

Acknowledgments: Dafna Greitzer-Antes provided the illustration for Supplementary Figure 9.

Supplementary Table 1. Flow cytometry human antibodies for HUCPVC characterization

**Antigen**	**Supplier**	**Dilution**
CD90-APC	R&D systems: (#130-114-903	1:40
CD105-APC	R&D systems: (#130-098-778	1:40
CD146-FITC	R&D systems: (#130-111-323)	1:40
HLA-G-FITC	R&D systems 130-112-004	1:40
PDGFR-β-APC	R&D systems # FAB1263A	1:20

Supplementary Table 2. Flow cytometry rat antibodies for EPC characterization

Antigen	Supplier	Dilution
CD31-FITC	R&D systems: (AF3628)	1:40
CD-34-FITC	R&D systems: (AF6518)	1:40
CD133-PE	Novus Biological (NB120-16518PE)	1:40
CD38-APC	Thermofisher 560846	1:20
VEGF-R2-PE	Novus Biological NB-200-208	1:20
CD146-FITC	BD Sciences 560846	1:20
VE-Cadherin-FITC	BioUSA BS-0878R-A488	1:20
CD117-APC	Tonbo Biosciences 20-1172-u025	1:20

SupplementaryTable 3. Primary antibodies for immunofluorescence staining

Antigen	Supplier	Host species	Dilution
Connexin-43	Abcam: ab11370	Goat	1:200
Sarcomeric actinin	Abcam: ab32575	Rabbit	1:200
PDGFR-β	Abcam: ab62437	Rabbit	1:200
Isolectin-GS-IB4	Life Technologies:1-21412	--	1:50
Thapsigargin	Life Technologies: B-7487	Goat	1:200
Cleaved caspase	Cell Signaling, 9661S	Rabbit	1:200
DQ Gelatin	Life Technologies	-	1:50

SupplementaryTable 4. Secondary antibodies for immunofluorescence staining (all purchased from Thermofisher)

Anti-rat IgG AF594	1:500
Anti-mouse IgG AF488	1:500
Anti-rabbit IgG AF647	1:500
Anti-goat IgG A-11055	1:500
Anti-sheep IgG A-11015	1:500

Supplementary Table 5: Custom Targeted RNA sequence Amplicon List

Amplicon Target	Aliases	Full Name	Chromosome/ Transcript	Start	Stop	Description
ANGPT1		Angiopoietin 1	NM_001146	973	1069	Angiogenic Factor
BDNF		Brain-Derived Neurotrophic Factor	NM_001143810	33	142	Developmental/ Pleiotrophic Factor
BMP1		Bone morphogenetic protein 1	NM_006129	2181	2283	Developmental/ Pleiotrophic Factor
BMP3		Bone morphogenetic protein 3	NM_001201	1479	1583	Developmental/ Pleiotrophic Factor
BMP4		Bone morphogenetic protein 4	NM_130851	579	686	Developmental/ Pleiotrophic Factor
BMP6		Bone morphogenetic protein 6	NM_001718	838	940	Developmental/ Pleiotrophic Factor
CCL11		C-C motif chemokine 11	NM_002986	265	368	Chemokine
CCL15	leukotactin-1, MIP5	C-C motif chemokine 15	NM_032965	753	858	Chemokine

CCL2	monocyte chemoattractant protein 1 (MCP1) and small inducible cytokine A2	C-C motif chemokine 2	NM_002982	243	347	Chemokine
CCL21		Chemokine C-C motif ligand 21	NM_002989	433	540	Chemokine
CCL26	Eotaxin-3	Chemokine (C-C motif) ligand 26	NM_006072	87	195	Chemokine
CCL5	RANTES	Chemokine (C-C motif) ligand 5	NM_002985	111	220	Chemokine
CNTF		Ciliary neurotrophic factors	NM_000614	95	199	Cytokine
CSF1	M-CSF	Colony-Stimulating Factor 1 or macrophage colony stimulating factor	NM_000757	807	903	Chemokine
CSF2	GM-CSF	Granulocyte-macrophage colony-	NM_000758	305	409	Chemokine

		stimulating factor				
CSF3		Granulocyte Colony-Stimulating Factor	NM_000759	152	256	Chemokine
CX3CL1	FRACTALKINE / neurotactin	C-X3-C motif ligand 1	NM_002996	191	299	Chemokine
CXCL1		C-X-C motif chemokine ligand 1	NM_001511	358	460	Chemokine
CXCL10	interferon gamma-induced protein 10	C-X-C motif chemokine ligand 10	NM_001565	355	460	Chemokine
SDF-1	SDF1, stromal cell-derived factor 1	C-X-C motif chemokine 12	NM_001178134	184	291	Chemokine
CXCL3		C-X-C motif chemokine 3	NM_002090	412	515	Chemokine
CXCL9		C-X-C motif chemokine 9	NM_002416	150	251	Chemokine
CXCR4	fusin, CD184	C-X-C chemokine	NM_003467	101	209	Chemokine Receptor

		receptor 4				
EDN1	ET-1, PPET1	Endothelin 1	NM_001955	549	655	Vasoregulator
EDN2	ET-2	Endothelin 2	NM_001956	193	304	Vasoregulator
EGF		Epidermal growth factor	NM_001963	167 5	178 4	Developmental/ Pleiotrophic Factor
EPO		Erythropoietin	NM_000799	397	503	Cytokine
ERAP1		Endoplasmic reticulum aminopeptidase 1	NM_016442	100 2	110 6	Aminopeptidas e
FGF1		Fibroblast growth factor 1	NM_000800	697	805	Developmental/ Pleiotrophic Factor
FGF2		Fibroblast growth factor 2	NM_002006	573	679	Developmental/ Pleiotrophic Factor
FGF23		Fibroblast growth factor 23	NM_020638	428	529	Developmental/ Pleiotrophic Factor
FGF5		Fibroblast growth factor 5	NM_004464	534	636	Developmental/ Pleiotrophic

						Factor
FGF6		Fibroblast growth factor 6	NM_020996	438	540	Developmental/ Pleiotrophic Factor
FGF7		Fibroblast growth factor 7	NM_002009	175	277	Developmental/ Pleiotrophic Factor
FIGF	VEGFD	Vascular endothelial growth factor D	NM_004469	909	101 3	Angiogenic Factor
FLT1		Vascular endothelial growth factor receptor 1	NM_002019	125 7	135 9	Angiogenic Factor Receptor
GDF10	BMP-3B	Growth Differentiation Factor 10	NM_004962	680	786	Cytokine
GDF11	BMP-11	Growth Differentiation Factor 11	NM_005811	445	554	Cytokine
GDF9		Growth Differentiation Factor 9	NM_005260	603	703	Cytokine

GDNF		Glial cell line - derived neurotrophic factor	NM_001190468	123	228	Developmental/ Pleiotrophic Factor
HBEGF		Heparin-binding EGF-like growth factor	NM_001945	733	835	Developmental/ Pleiotrophic Factor
HGF		Hepatocyte Growth Factor	NM_000601	495	596	Developmental/ Pleiotrophic Factor
HMOX1		Heme Oxygenase-1	NM_002133	638	745	Regulator of paracrine activity
IDO1		Indoleamine-2,3-dioxygenase	NM_002164	269	370	Regulator of paracrine activity
IGF1		Insulin-like growth factor 1	NM_001111285	418	519	Developmental/ Pleiotrophic Factor
IGF2		Insulin-like growth factor 2	NM_001127598	558	664	Developmental/ Pleiotrophic Factor
IGFBP2		Insulin like growth factor binding protein 2	NM_000597	894	1002	Developmental/ Pleiotrophic Factor

IL10		Interleukin-10	NM_000572	492	599	Cytokine
IL11	AGIF, adipogenesis inhibitor factor	Interleukin-11	NM_000641	354	452	Cytokine
IL13		Interleukin-13	NM_002188	269	379	Cytokine
IL18		Interleukin-18	NM_001562	141	247	Cytokine
IL18BP		Interleukin-18-binding protein	NM_001039660	189	293	Cytokine
IL1A		Interleukin-1A	NM_000575	863	969	Cytokine
IL1B		Interleukin-1B	NM_000576	660	767	Cytokine
IL1R1		Interleukin 1 receptor type 1	NM_000877	640	743	Cytokine Receptor
IL1R2		Interleukin 2 receptor type 2	NM_004633	222	332	Cytokine Receptor
IL4		Interleukin-4	NM_000589	381	478	Cytokine
IL4R		Interleukin 4 receptor	NM_000418	449	557	Cytokine Receptor
IL6		Interleukin-6	NM_000600	522	627	Cytokine

IL6R		interleukin-6 receptor	NM_000565	830	936	Cytokine Receptor
IL7		Interleukin-7	NM_000880	546	653	Cytokine
JAG1		Jagged-1	NM_000214	2157	2265	Developmental/ Pleiotrophic Factor
JAG2		Jagged-2	NM_145159	2838	2940	Developmental/ Pleiotrophic Factor
JUN	AP-1 Transcription Factor subunit	Jun Proto-Oncogene	NM_002228	49	153	housekeeping gene
KDR	VEGFR-2, vascular endothelial growth factor receptor 2	Kinase insert domain receptor	NM_002253	1486	1587	Angiogenic Receptor
KITLG	SCF, stem cell factor	Kit Ligand	NM_000899	150	250	Developmental/ Pleiotrophic Factor
LEFTY2	TGF-B4, Transforming Growth Factor 4	Left-Right Determination Factor	NM_003240	430	529	Developmental/ Pleiotrophic Factor

LGALS3	GAL-3	Galectin-3	NM_002306	375	483	Developmental/ Pleiotrophic Factor
LGALS9	GAL-9	Galectin-9	NM_009587	74	176	Developmental/ Pleiotrophic Factor
LIF		Leukemia Inhibitory Factor	NM_002309	302	409	Developmental/ Pleiotrophic Factor
MBTPS1		Membrane-bound transcription factor peptidase, site 1	NM_003791	1284	1385	Cell surface Protease
MDK	Neurite Growth-Promoting Factor 2	Midkine	NM_001012334	563	659	Developmental/ Pleiotrophic Factor
MMP1		Matrix metalloproteinase-1	NM_002421	690	798	Cell surface Protease
MMP2		Matrix metalloproteinase-2	NM_004530	1106	1214	Cell surface Protease

MMP9		Matrix metalloproteinase-9	NM_004994	1175	1284	Cell surface Protease
MRPL13		Mitochondrial Ribosomal Protein L13	NM_014078	238	342	housekeeping gene
NGF		Nerve Growth Factor	NM_002506	119	225	Developmental/ Pleiotrophic Factor
NOS2		Nitric Oxide Synthase 2	NM_000625	92	196	Regulator of paracrine activity
NPPB		Natriuretic Peptide B	NM_002521	424	534	Hormone
NRG1		Neuregulin 1	NM_013956	700	802	Developmental/ Pleiotrophic Factor
NTF3	NT-3	Neurotrophin 3	NM_001102654	195	299	Neurotrophic Factor
PDGF-A		Platelet-derived growth factor A	NM_002607	1202	1308	Developmental/ Pleiotrophic Factor
PDGF-B		Platelet-derived growth factor B	NM_002608	1711	1817	Developmental/ Pleiotrophic Factor

PDGFC		Platelet-derived growth factor c	NM_016205	1314	1422	Developmental/ Pleiotrophic Factor
PDGFR-A		Platelet-derived growth factor receptor alpha	NM_006206	1196	1302	Receptor
PDGFRB		Platelet-derived growth factor receptor beta	NM_002609	2794	2902	Receptor
PECAM1		Platelet and Endothelial Cell Adhesion Molecule	NM_000442	23	133	Cell adhesion Molecule
PF4		Platelet factor 4	NM_002619	165	273	Chemokine
PGF		Placental Growth Factor	NM_002632	743	843	Developmental/ Pleiotrophic Factor
PLAT	Tissue-type plasminogen activator; TPA	Plasminogen activator	NM_000930	1652	1762	Angiogenic Factor
PLG		Plasminogen	NM_000301	66	165	Angiogenic Factor
PTGES2		Prostaglandin E synthase 2	NM_025072	1423	1530	Regulator of paracrine

						activity
PTGS1	Cyclooxygenase-1 (COX1)	Prostaglandin-Endoperoxide Synthase 1	NM_000962	675	782	Regulator of paracrine activity
PTGS2	Cyclooxygenase 2 (COX2)	Prostaglandin-Endoperoxide Synthase 2	NM_000963	1477	1584	Regulator of paracrine activity
SERPINE1	Serpin E1, Plasminogen Activator Inhibitor Type 1	Serpin Family E Member 1)	NM_000602	1099	1211	Angiogenic Factor
SPP1	Osteopontin	Secreted Phosphoprotein 1	NM_001040058	602	711	Cytokine
STC1		Stanniocalcin-1	NM_003155	755	862	hormone
TDGF1		Teratocarinoma-derived growth factor 1	NM_001174136	431	525	Developmental/ Pleiotrophic Factor
TGF-B1		Transforming Growth Factor beta 1	NM_000660	1369	1475	Developmental/ Pleiotrophic Factor
THBS1		Thrombospondin	NM_003246	141	151	Adhesion

		1		2	7	protein
TIMP1		Tissue inhibitor of metalloproteinases	NM_003254	368	472	Metalloproteinase inhibitor
TNF		Tumor necrosis factor	NM_000594	451	556	Cytokine
TNFAIP6		Tumor Necrosis Factor, Alpha-Induced Protein 6	NM_007115	217	327	Regulator of paracrine activity
TNFRSF1A		Tumor Necrosis Factor Receptor 1	NM_001065	463	566	Cytokine Receptor
TNNT1		Troponin T1	NM_001126132	335	446	Other
PECAM1	ECGF1	Thymidine Phosphorylase	NM_001113756	874	978	Regulator of paracrine activity
VEGFA		Vascular Endothelial Growth Factor A	NM_001171623	1281	1390	Angiogenic Factor
VEGFC		Vascular Endothelial	NM_005429	1484	1591	Angiogenic Factor

		Growth Factor C				

1

2

3

SUPPLEMENTARY FIGURES 1-15

Supplementary Figure 1. Cell retention of FTM HUCPVC in combination treatments following 5-days post cell injections (5 Days).

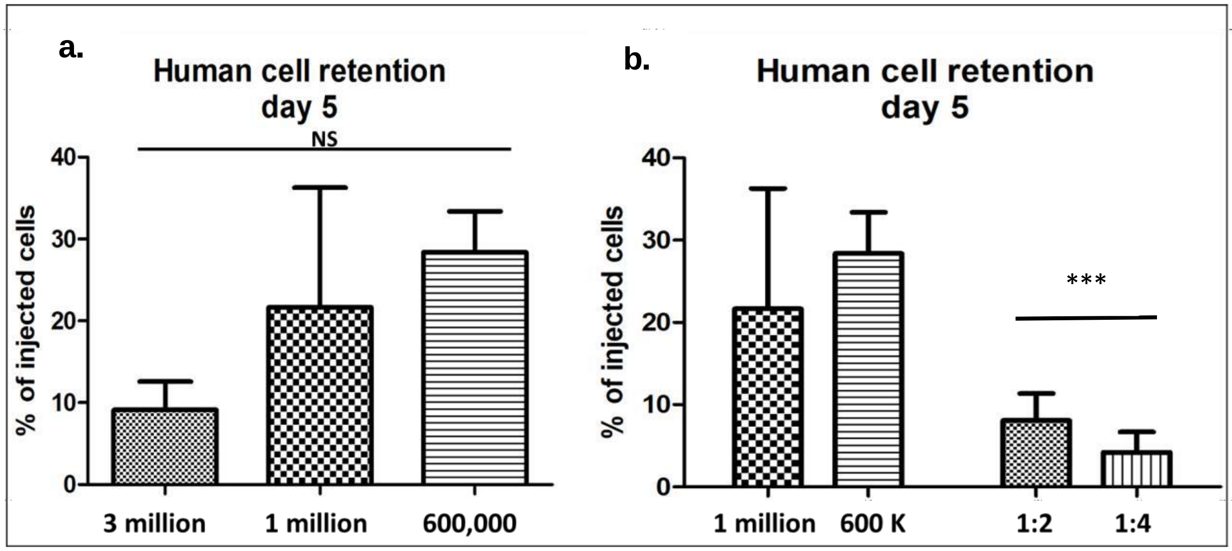

Supplementary Figure 1: (a) Relative cell retention based on Alu qPCR Ct values of FTM HUCPVC injected hearts at Day 5 normalized to FTM HUCPVC injected hearts at Day 0. Greater cell retention was observed in 600K and 1 million injected FTM HUCPVC compared to 3 million injected treatment group. **(b)** Significantly lower human cell retention was observed in combination treatment groups (*p*<0.001) compared to 600K and 1 million FTM HUCPVC injected hearts. Ct<28 was considered. Statistical analysis by One-Way ANOVA using Tukey post-hoc analysis. * p<0.05, *p<0.01, *** p<0.001. N=3.

Supplementary Figure 2. Fluorescence imaging of human-PDGFR-β positive signal co-localized with myocardial vasculature (CD31) (5 Days).

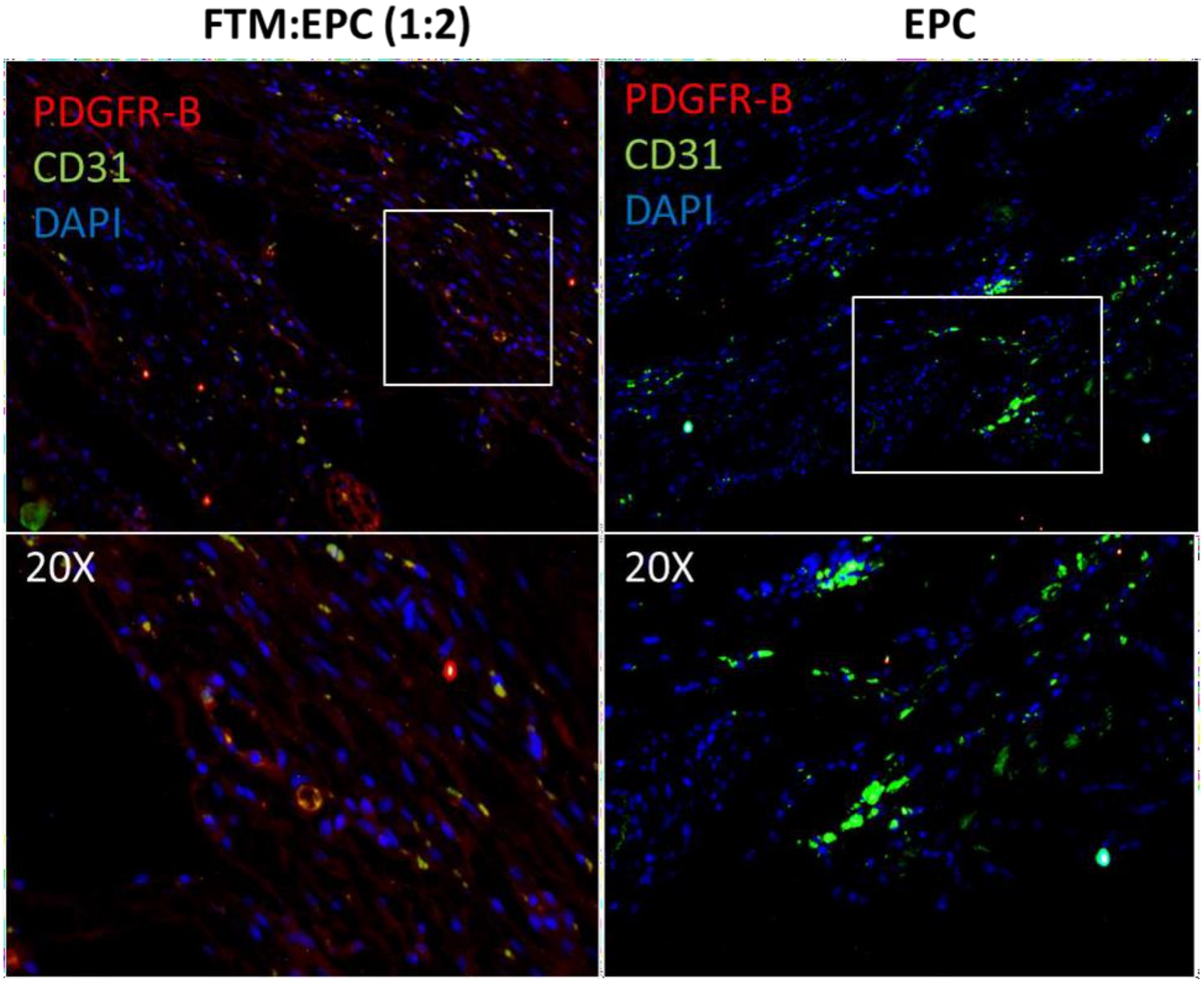

Supplementary Figure 2. Heart sections were stained using human specific antibody for PDGFR-β(red) and rat-specific antibody for endothelial cells (CD31). High magnification images show overlapping signal of PDGFR-β and CD31 in 1:2 combination treated hearts, where no PDGFR-B signal was detected in ECFC-only injection group. These results suggest close association of pericytes with rat myocardial vasculature following FTM HUCPVC and ECFC co-injection. Scale bar: 500µm. N=3.

Supplementary Figure 3: FTM HUCPVCs and ECFCs retain in the LV following 5-days post MI, limited changes in circulating rat-derived inflammatory factors

a.

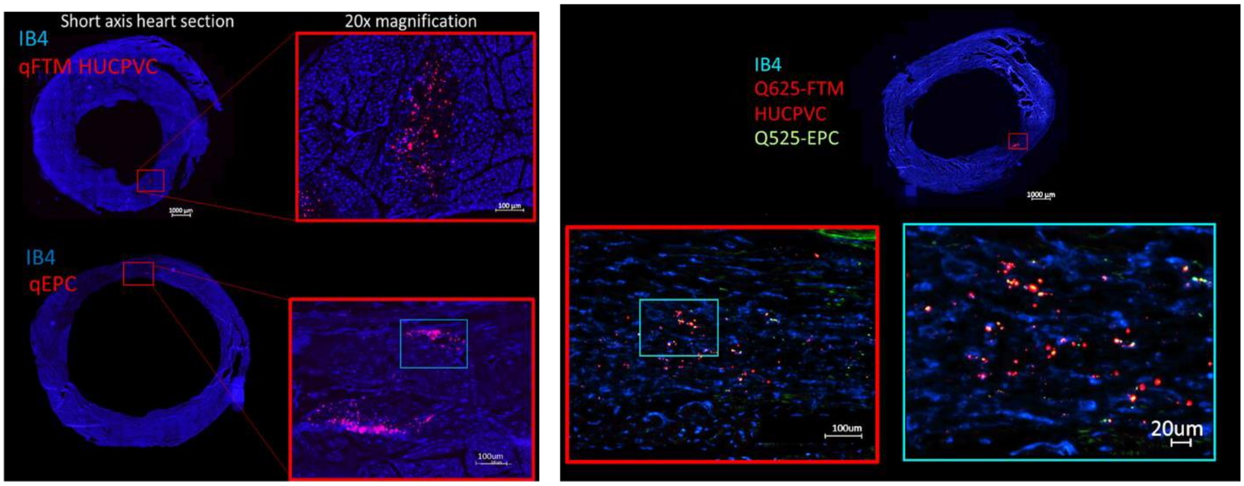

b.

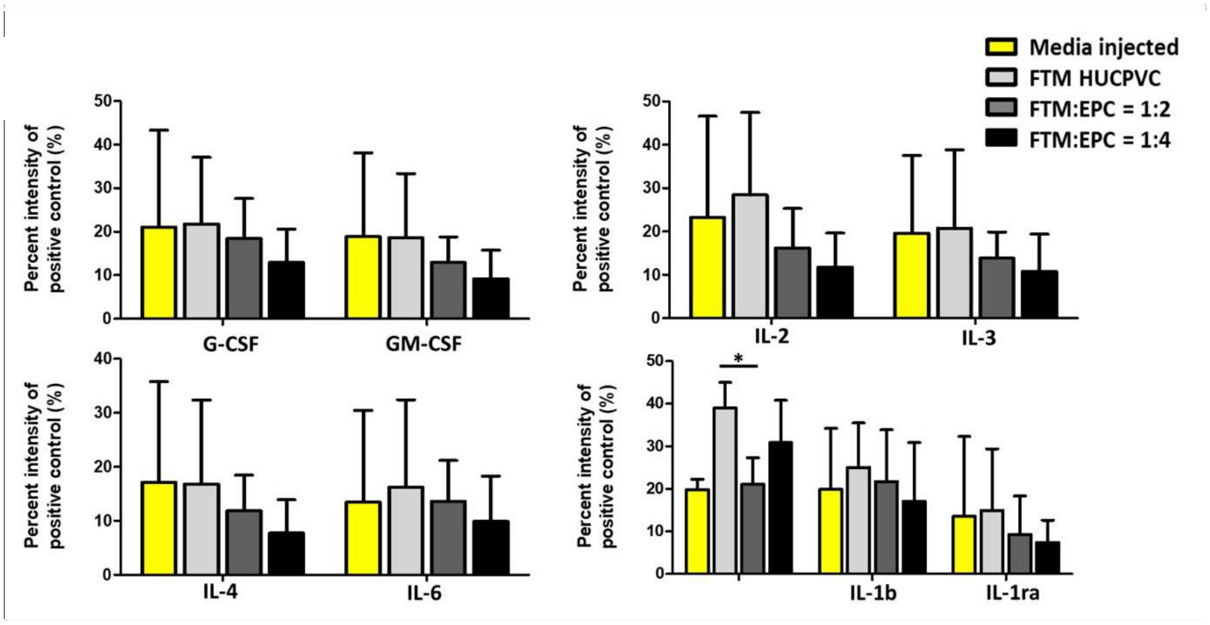

c.

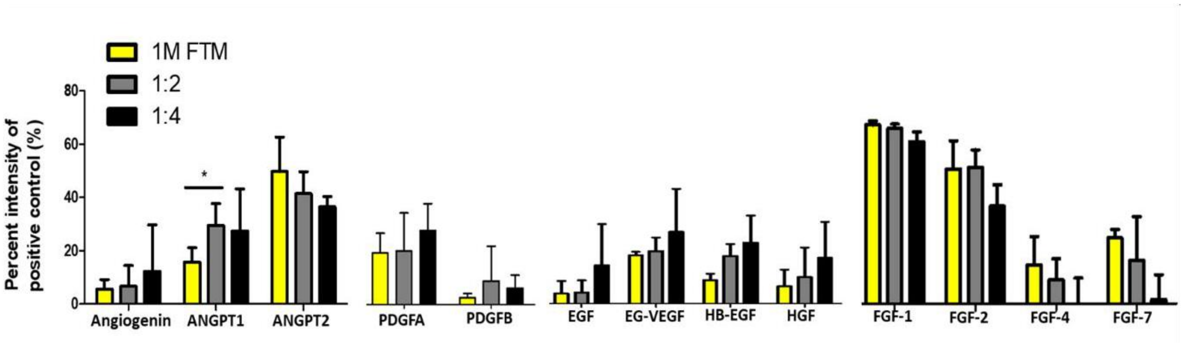

Supplementary Figure 3: (a) Immunofluorescence imaging of pre-labelled FTM HUCPVCs and ECFCs post 5-day injection following MI. Five days following injection, FTM HUCPVCs and ECFCs individually (red) were detected in the left ventricle of MI hearts. Pre-labeled FTM HUCPVC and ECFC combinations were co-localized (yellow signal) within the LV. **(b)** Quantification of rat-specific protein in the serum were quantified for immunomodulatory factors and cytokines following cell therapy. No significant changes were detected following cell therapy, whereas a significant reduction in IL-1a ($p<0.05$) was detected in the 1:2 combination treatment group (N=3). **(c).** Quantification of human pro-angiogenic factors in rat serum. No significant differences were detected except for a significant increase in ANGPT1 in 1:2 combinations compared to FTM alone ($p<0.05$). Statistical analysis by One-Way ANOVA using Tukey post-hoc analysis. * $p<0.05$, *$p<0.01$, *** $p<0.001$. Medium injection serves as positive control. Y axis: normalized to positive loading control, X axis: different treatment groups.

Supplementary Figure 4. Pressure volume loops of cell therapy treatments at 4 weeks post injection.

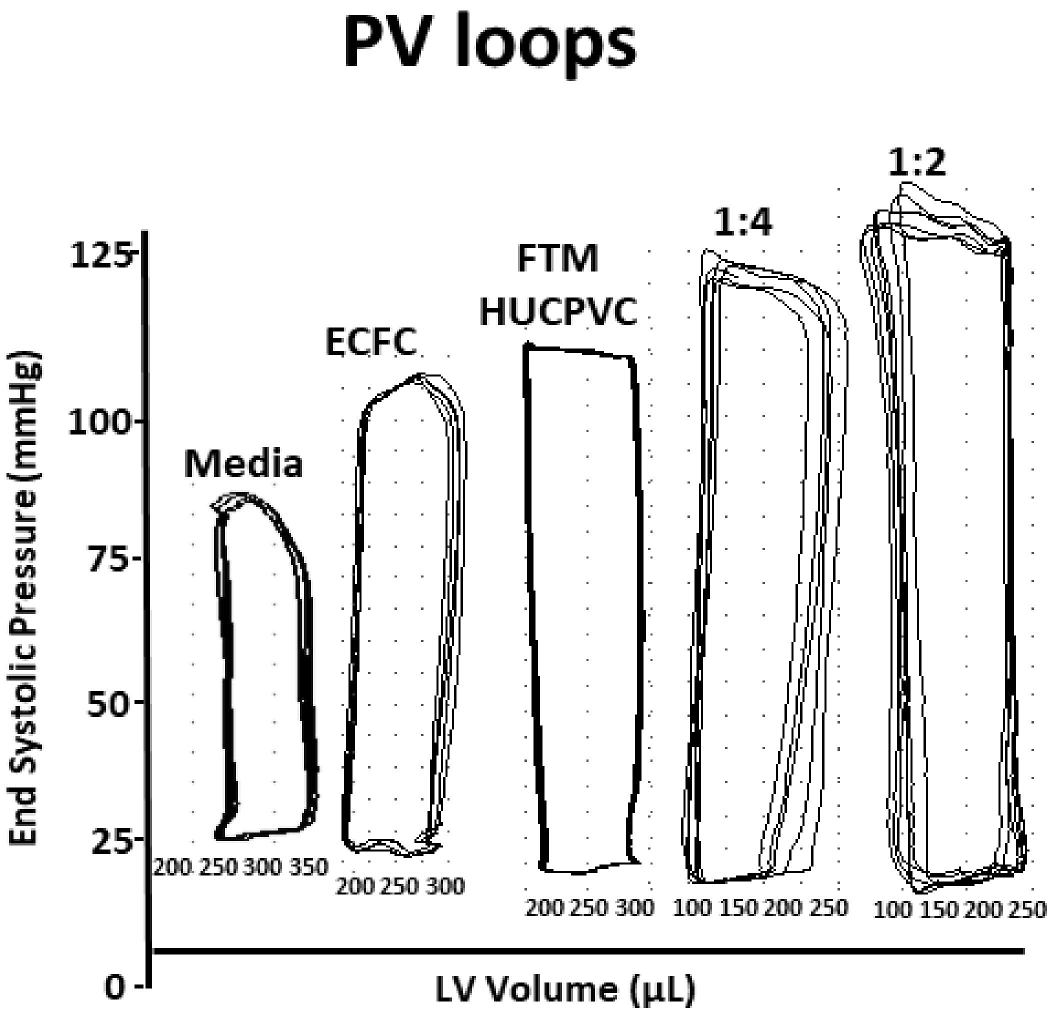

Supplementary Figure 4: The panel shows representative cardiac performance loops recorded for each of the 5 groups at 28 days after treatment. Declining cardiac function is represented by a smaller end systolic pressure and larger LV volumes. N=9-12.

Supplementary Figure 5. Morphological and phenotypical analysis of FTM HUCPVC.

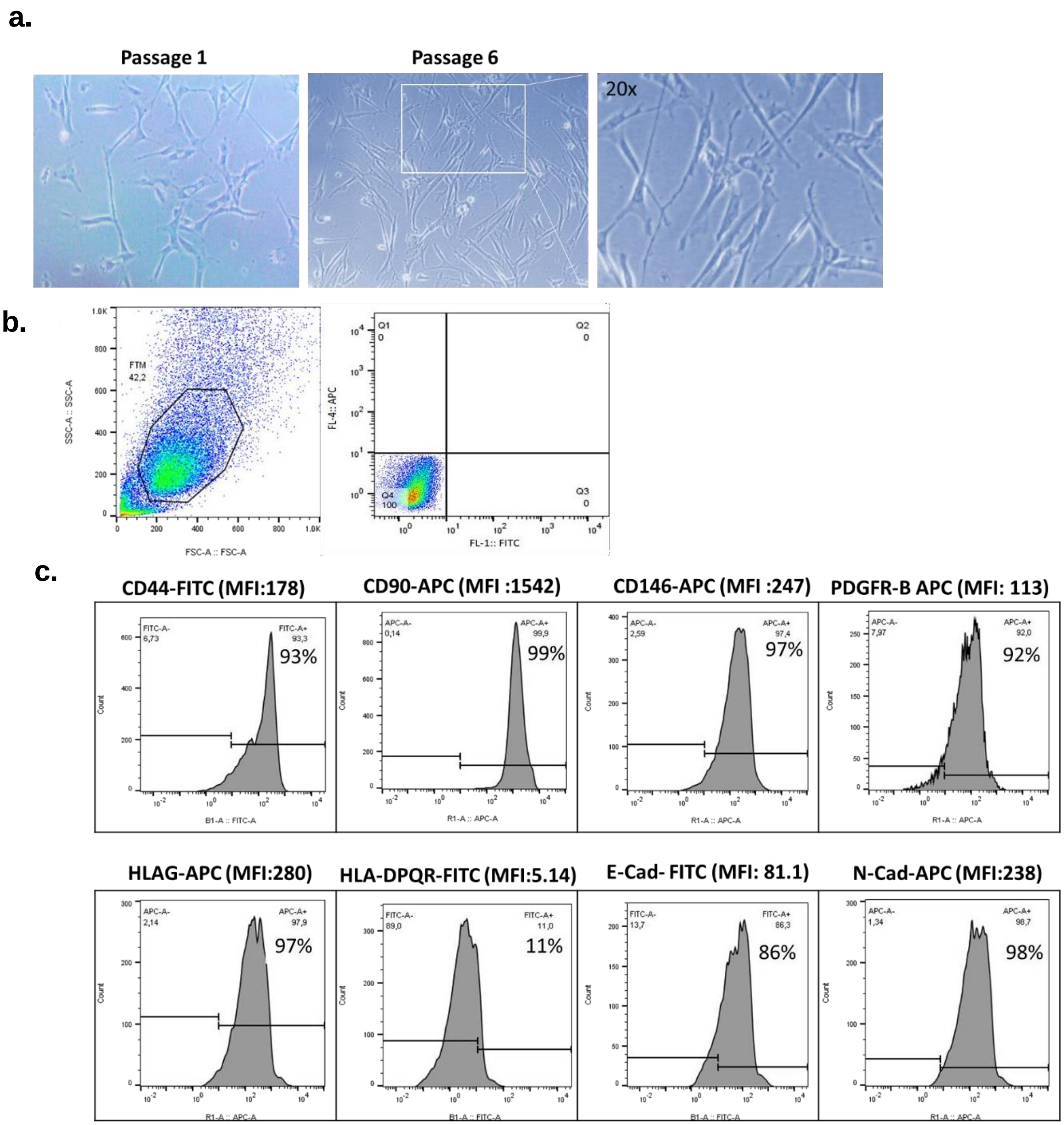

Supplementary Figure 5: (a) Bright field microscopy images of FTM HUCPVC at passage 1 and passage 3. FTM HUCPVC demonstrate fibroblast-like morphologies with spindle-like processes. **(b)** Gating strategy for flow cytometry analysis of FTM HUCPVC including all cells and negative controls. **(c)** Histograms depicting phenotypes of HUCPVC and pericyte-associated markers. HUCPVC markers CD44, CD90; pericyte-makers including CD146,

PDGFR-B, E-cadherin N-cadherin; inflammation associated markers include HLA-G, HLA-DP, DQ, DR. Gates on histograms are determined from unstained FTM HUCPVC, where mean MFIs are indicated for each associated marker. Percentages represent positive events within total gated cells. FTM HUCPVC are positive for MSC and pericyte-associated phenotypic markers. N=3.

Supplementary Figure 6: Pericyte-like phenotypes of HUCPVC.

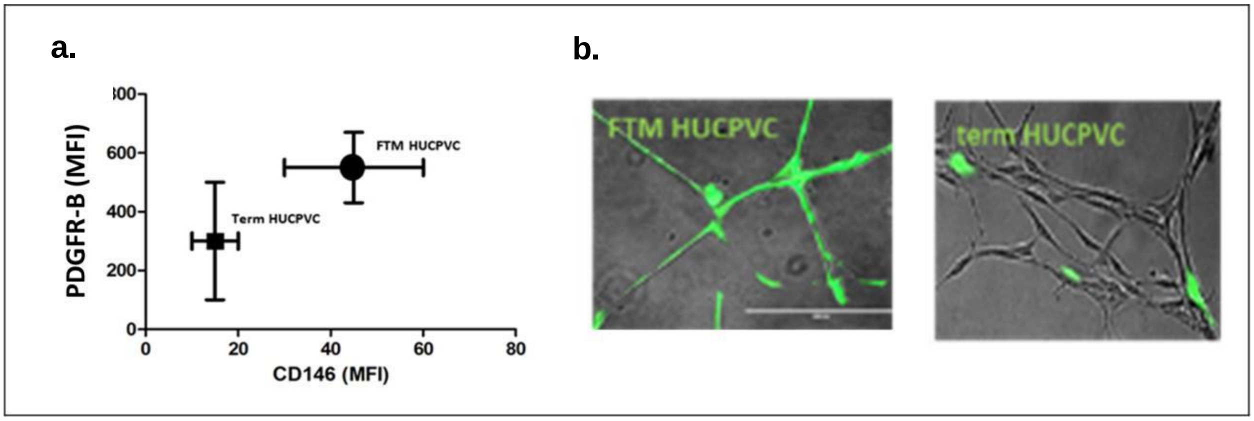

Supplementary Figure 6: (a) Flow cytometry analysis of pericyte markers including, PDGFR-B and CD146, expressed as average mean fluorescent intensities (MFI). Greater MFIs were observed for both PDGFR-β and CD146 in cultured FTM HUCPVC compared to term HUCPVC. **(b)** Pre-labelled HUCPVC were co-cultured with aortic ring-derived endothelial networks and fluorescence imaging identified HUCPVC-mediated endothelial network coverage. Greater homing and network integration were observed in FTM HUCPVC treated co-cultures compared to term HUCPVC co-cultures. Scale: 200µm. N=4. The MFI data in the graph is a sum of multiple runs performed therefore individual gates not shown. This image has been published previously by our team in a book chapter[1].

Supplementary Figure 7: Phenotypic Characterization of Endothelial Progenitor Cells

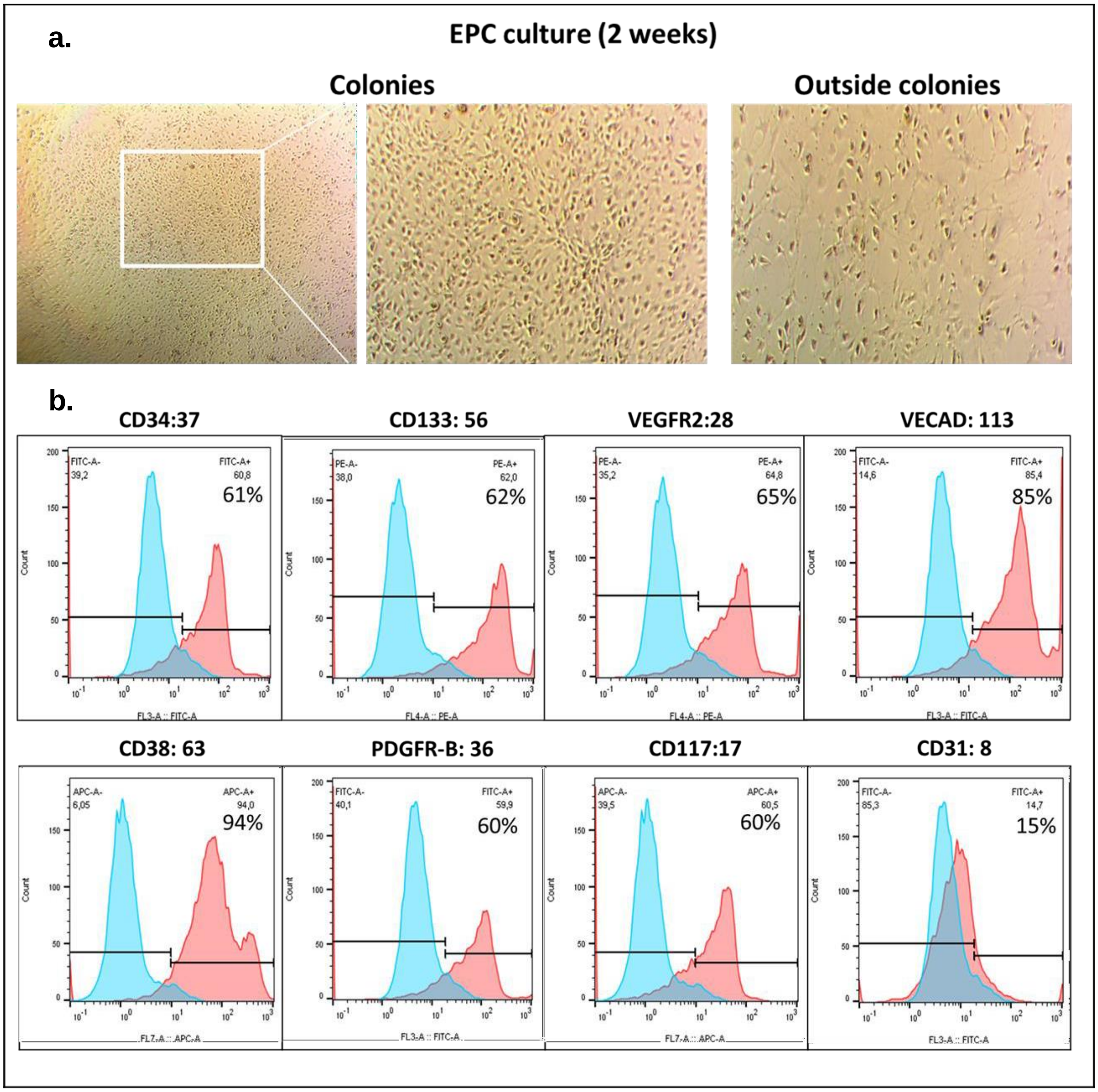

Supplementary Figure 7: (a) Bright field images of ECFC cultured in T75 flasks. At 14-days of cell culture, cobblestone shaped ECFC were observed in large colonies throughout culture flasks. Spindle shaped ECFC were identified outside ECFC colonies. **(b)** Flow cytometry analysis of ECFC 14-days post culture depicted in histograms. Blue plots represent isotype control populations, where red plots represent positive populations. Mean fluorescence intensities of corresponding markers are indicated on top of the histograms. Percentage values

represents positive gated cells compared to negative control. ECFC demonstrated high positivity for progenitor-associated markers (CD34 and CD133); high positivity for endothelial lineage-associated markers (VEGFR2, VECAD) and low positivity for CD31 (mature EC marker). y axis: cell counts, x axis: mean fluorescence intensity. N=3.

Supplementary Figure 8: Comparison of early and late ECFC phenotype profiles.

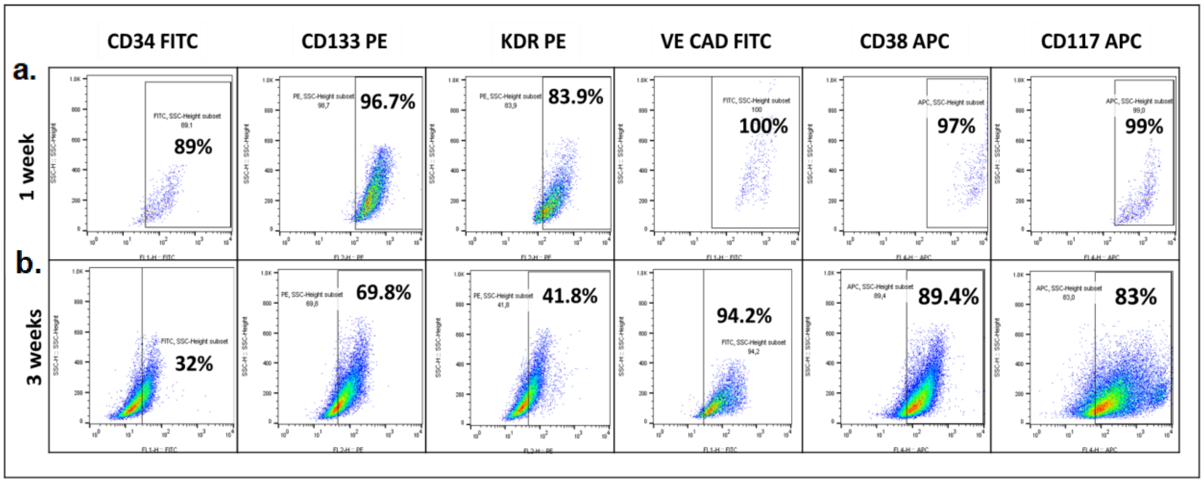

c.

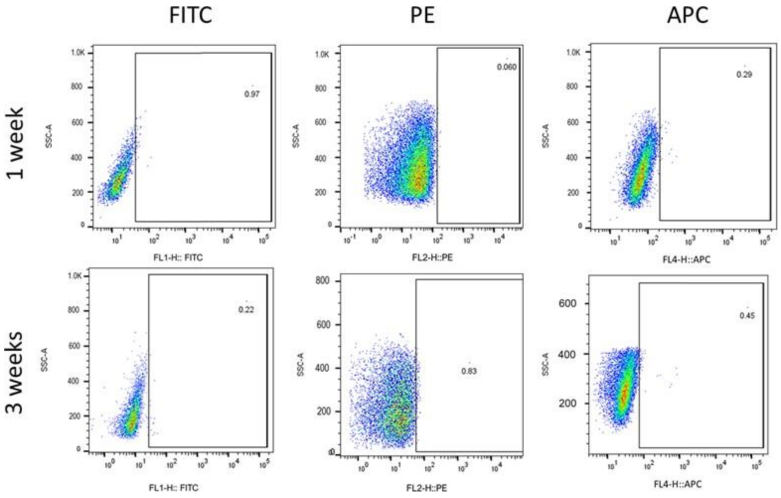

Supplementary Figure 8: (a) Flow cytometry analysis of rat bone marrow ECFC harvested at 1-week of culture. Scatter plots include gated ECFC populations based on respective isotype controls. Percentages represent positive gated ECFC compared to respective isotype controls. Early ECFC were highly positive for progenitor markers including CD34+ (89%) and CD133+ (97%) and highly positive for KDR (84%) and VECAD (100%). **(b)** ECFC harvested at 3-weeks following culture demonstrated down-regulation of progenitor markers including CD34+ (32%) and CD133+ (70%) and endothelial markers including KDR+ (42%) and VECAD (94%). ECFC

were used at 2 weeks for experiments. **(C)** Controls for each condition and timepoint. y axis: side scatter height, x axis: fluorescence intensity. N=3.

Supplementary Figure 9: Schematic of *in vitro* setup for HUCPVC ad ECFC co-culture.

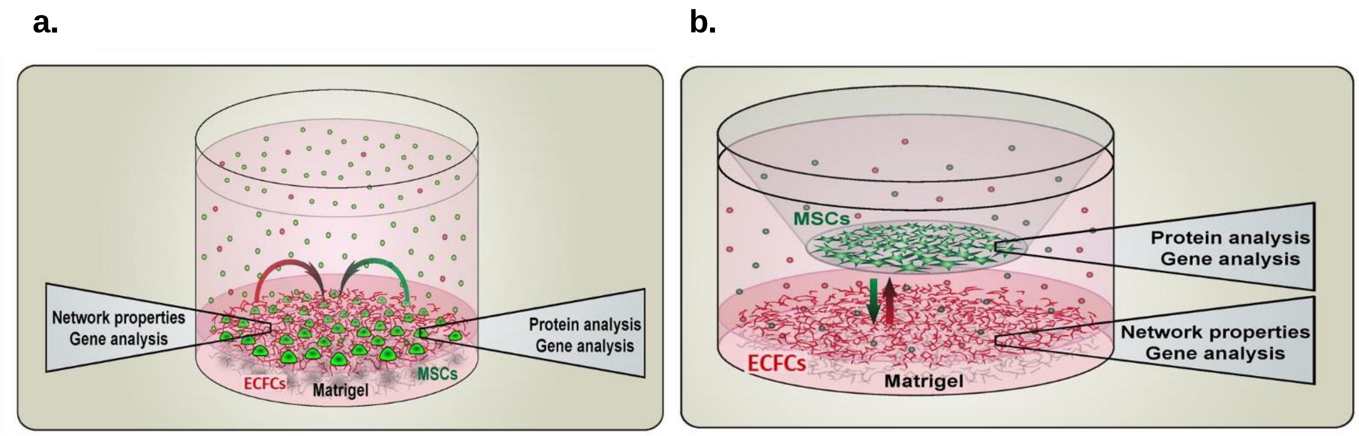

Supplementary Figure 9: Schematics of *in vitro* angiogenesis assays. (a). Direct co-culture of HUCPVCs and ECFCs on Matrigel coated well. **(b).** Transwell co-culture of HUCPVCs and ECFCs. HUCPVCS were seeded onto transwell inserts and ECFCs cultured on Matrigel coated wells.

Supplementary Figure 10: Protein array analysis of conditioned medium from HUCPVC-ECFC transwell and direct co-cultures.

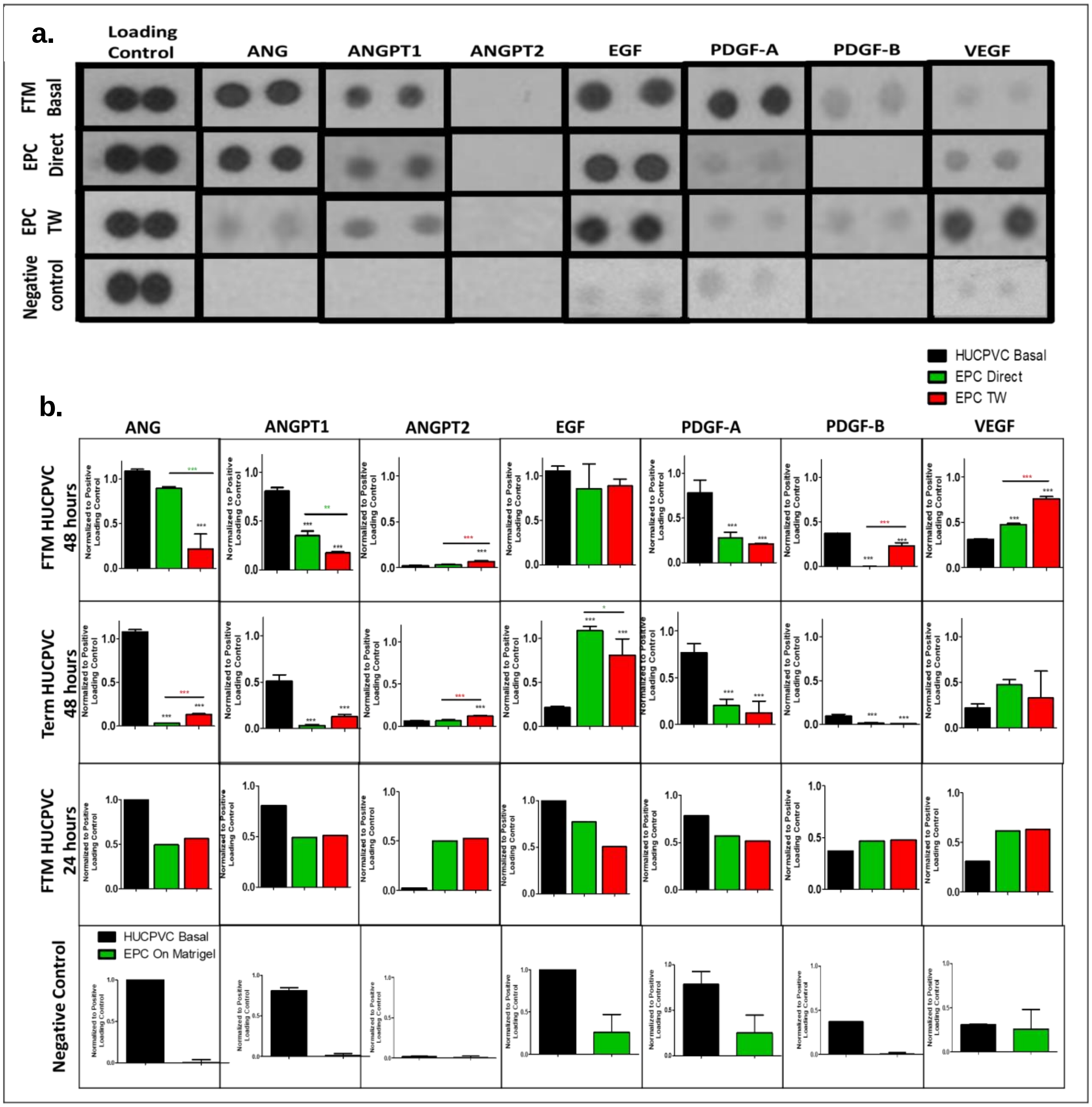

Supplementary Figure 10: (a) Dot plots represent specific proteins detected in FTM HUCPVC basal and ECFC co-culture conditioned medium (48 hours). (**b**). Significantly higher levels of angiogenin and ANGPT1 were detected in FTM HUCPVC-ECFC direct co-cultures compared to TW co-cultures ($p<0.01$). Significantly lower levels of angiogenin and ANGPT1 were detected in term HUCPVC-ECFC co-cultures ($p<0.001$) ($N=3$). Conditioned medium collected at 24 hours

from FTM HUCPVC-ECFC direct co-cultures were positive for many pro-angiogenic growth factors, comparable to basal FTM HUCPVC conditioned medium values (bottom row). (N=2). Statistical analysis by One-Way ANOVA. * $p<0.05$, *$p<0.01$, *** $p<0.001$. Y axis: Normalized protein level to internal positive loading control X axis: Treatment (Basal levels-black, direct co-culture levels-green and transwell co-culture levels-red). Black asterisks compare between proteins levels from specific treatment to basal HUCPVC-derived protein levels. Negative control includes ECFC cultured alone on Matrigel without HUCPVC.

Supplementary Figure 11: ECFC express pro-angiogenic factors that are maintained following FTM HUCPVC coculture

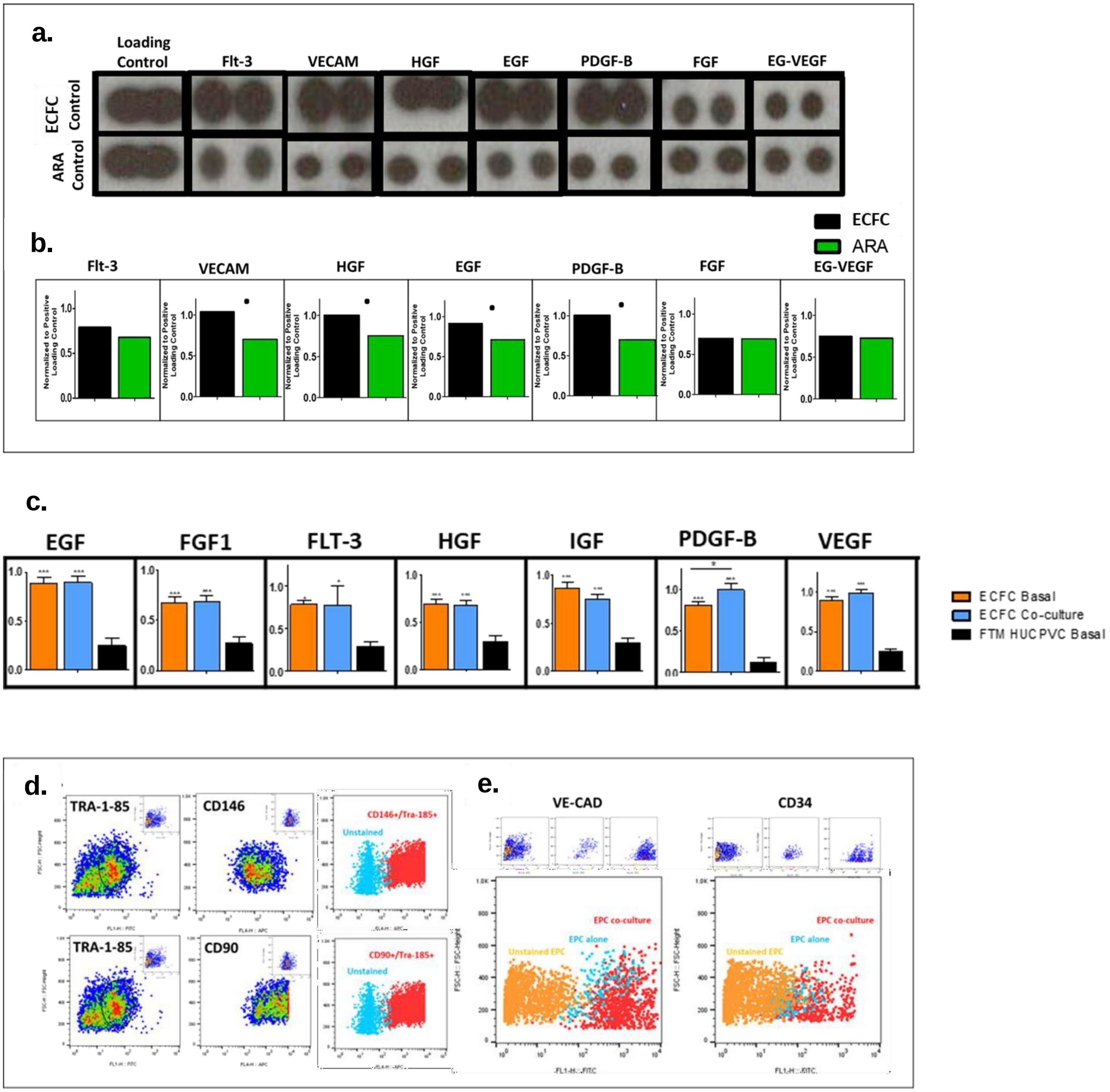

Supplementary Figure 11: (a) Dot plots represent specific protein intensities detected in ECFC only and aortic ring assay control conditioned medium (24 hours). **(b)** Higher levels of pro-angiogenic factors (FLT-3, VECAM, HGF, EGF, PDGF-B) detected in ECFC cultures compared to aortic ring assay (mature aortic-derived endothelial cells). (No statistics, n=2). Y axis:

Normalized protein level to internal positive loading control X axis: Treatment (ECFC control levels-black, aortic ring assay control levels-green). Black dots above bar graphs represent differences larger than 0.2 between treatment groups. **(c)** Rat angiogenesis array (R&D). Conditioned medium collected at 24 hours following co-culture with FTM HUCPVCs. FTM HUCPVCs serves as a negative control. ECFCs in co-culture with FTM HUCPVCs, increase levels of PDGF-β compared to ECFC single cell cultures (p<0.05). **(d)** MSC marker CD90 and pericyte marker CD146 were assessed using human specific primary flow antibodies following ECFC co-culture. Scatter plots show positivity of CD146+ and CD90+ which were gated for TRA-185+ FTM HUCPVCs. Tp right corners show scatter plot of respective isotype controls. FTM HUCPVCs maintain pericyte (CD146) and MSC (CD90) phenotypes following ECFC co-culture (red dots). **(e)** Scatter plots show merged plots of unstained ECFCs (yellow dots), ECFCs from single cultures (blue dots) and ECFCs from direct FTM HUCPVCs co-cultures (red dots). ECFCs from direct co-cultures up-regulated the expression of VE-Cadherin and CD34 compared to ECFCs cultured alone. Unstained negative controls are documented above panel e, (top left). N=3. Y axis: Forward scatter (height) X axis: Mean fluorescence intensity.

Supplementary Figure 12: Next generation sequencing analysis of term HUCPVC following 5 days of co-culture with ECFC in transwell or direct co-cultures.

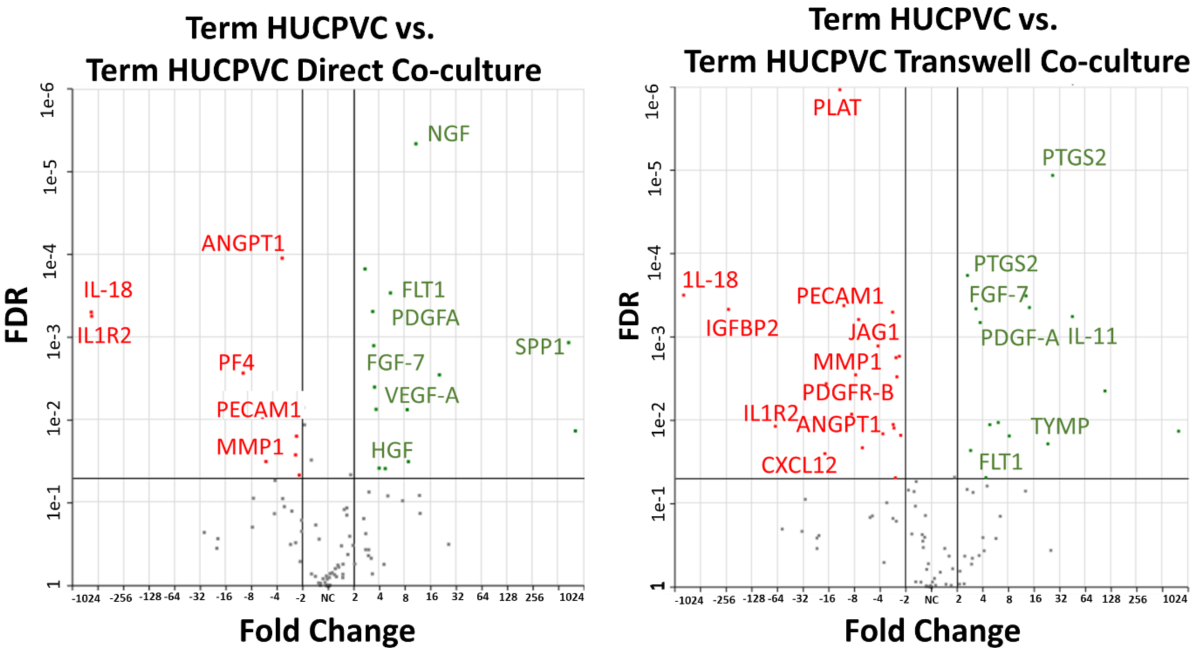

Supplementary Figure 12: (a) Volcano plot showing significant differential gene expression (highlighted by red) of term HUCPVC following co-culture with ECFC compared to term HUCPVC from basal culture conditions (green). Statistical analysis by One-Way ANOVA. Y axis of volcano plots: FDR (adjusted *p* value), X axis of volcano plots: fold change. Y axis of bar graphs: transcript counts. N=3.

Supplementary Figure 13: Confirmation of siRNA silencing of PDGFR-β and CD146 by flow cytometry and qPCR.

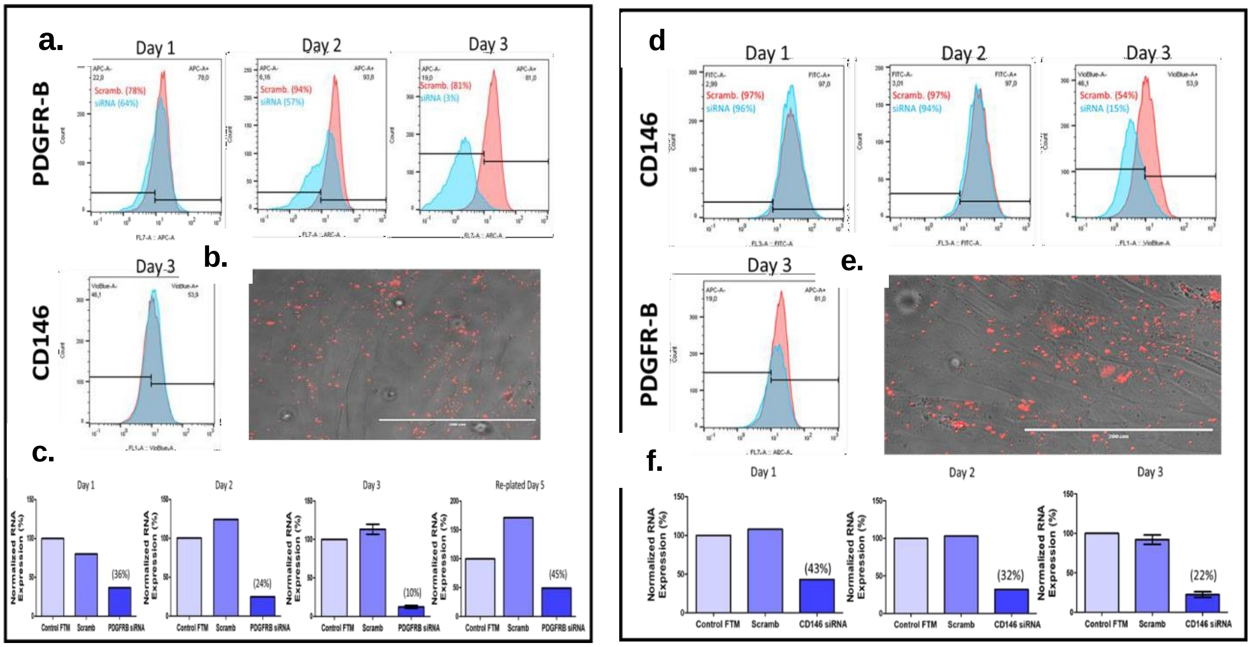

Supplementary Figure 13: (a) Flow cytometry of FTM HUCPVC following PDGFR-β-targeting siRNA transfection (Day 1-3). FTM HUCPVC down-regulated PDGFR-β to 3% positivity (blue) compared to 81% positivity in scrambled siRNA controls (red). No effects on CD146 expression were observed. N=3 **(b)** Fluorescence imaging of FTM HUCPVC co-cultured with BlockIt™ and PDGFR-β -siRNA to assess transfection efficiency. **(c)** qPCR of FTM HUCPVC following *PDGFR*-β-targeted siRNA transfection. *PDGFR*-β is down-regulated by 90% compared to scrambled siRNA and basal FTM HUCPVC control (Day 3). FTM HUCPVC re-plated for 5 days following PDGFR-β silencing up-regulated *PDGFR-B* by 35% compared to day 3 of transfection. N=2 **(d)** FTM HUCPVC down-regulated CD146 to 15% positivity compared to 54% positivity in scrambled controls. No effects on PDGFR-β expression were observed. N=3 **(e)** Fluorescence imaging of FTM HUCPVC incubated with BlockIt™ and CD146-siRNA to assess transfection efficiency. **(f)** *CD146* was down-regulated by roughly 80% compared to scrambled and basal FTM HUCPVC control (Day 3) N=2. Flow cytometry histograms (a, d) overlays of scrambled treatment (red) and PDGFR- β/CD146 transfection treatment (blue). Isotype control identified by

blue plot. See Supplementary Figure 14 for cell events. Y axis: cell counts, x axis: MFI of associated marker. Scale bar: 200µm.

Supplementary Figure 14: FTM HUCPVCs maintain mesenchymal stromal cell phenotype following silencing PDGFR-β

a.

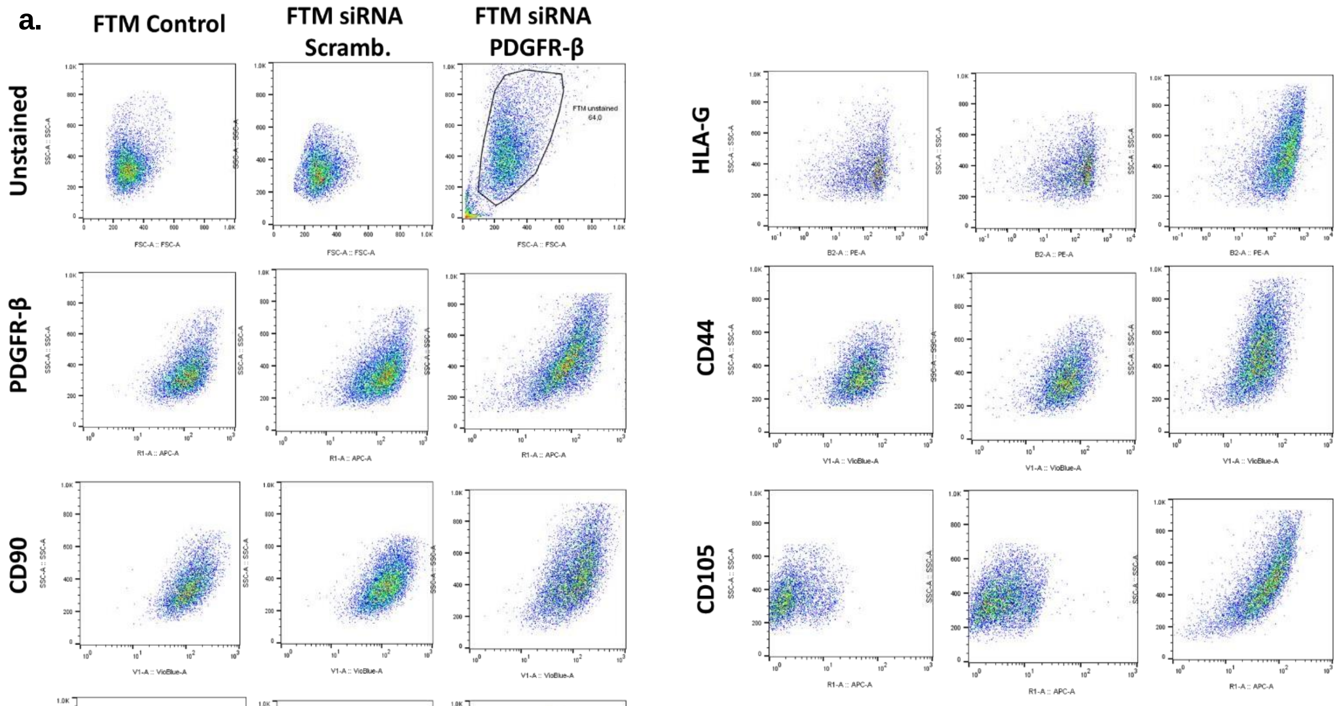

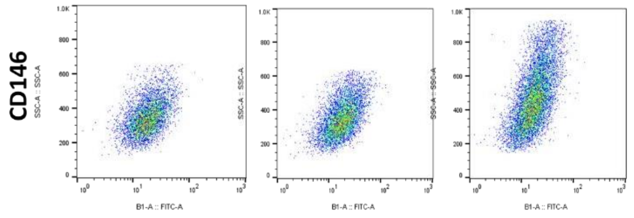

b.

Antibody	FTM HUCPVC Control	FTM siRNA scrambled unstained	FTM siRNA PDFGR-β
PDGFRb-APC	115	129	87,6
CD 90 Vioblue	114	120	112
CD 146 FITC	18	17,8	17,4
HLAG-PE	194	176	301
CD44 Vioblue	48,1	53,2	46,8
CD105 APC	2,21	3,80	56,8

Supplementary Figure 14: Immunophenotypic analysis of FTM HUCPVCs following the silencing of PDGFR-β. **a.** Mean florescent intensity (MFI) plots highlighting the expression of pericytes markers (PDGFR-β, CD146), mesenchymal stromal cell markers (CD90, CD150), immunoprivileged marker (HLA-G) and progenitor phenotype (CD44) of FTM HUCPVCs with no siRNA, FTM HUCPVC with siRNA scrambled and siRNA PDGFR-β. **b.** Table identifying MFIs of specific markers per culture condition. Analysis conducted three days following siRNA PDGFR-β. This data includes mean florescent intensities and did not include positive or negative gating.

Supplementary Figure 15: Fluorescence imaging of FTM HUCPVC co-cultured with ECFC following PDGFR-β neutralization (24 hours).

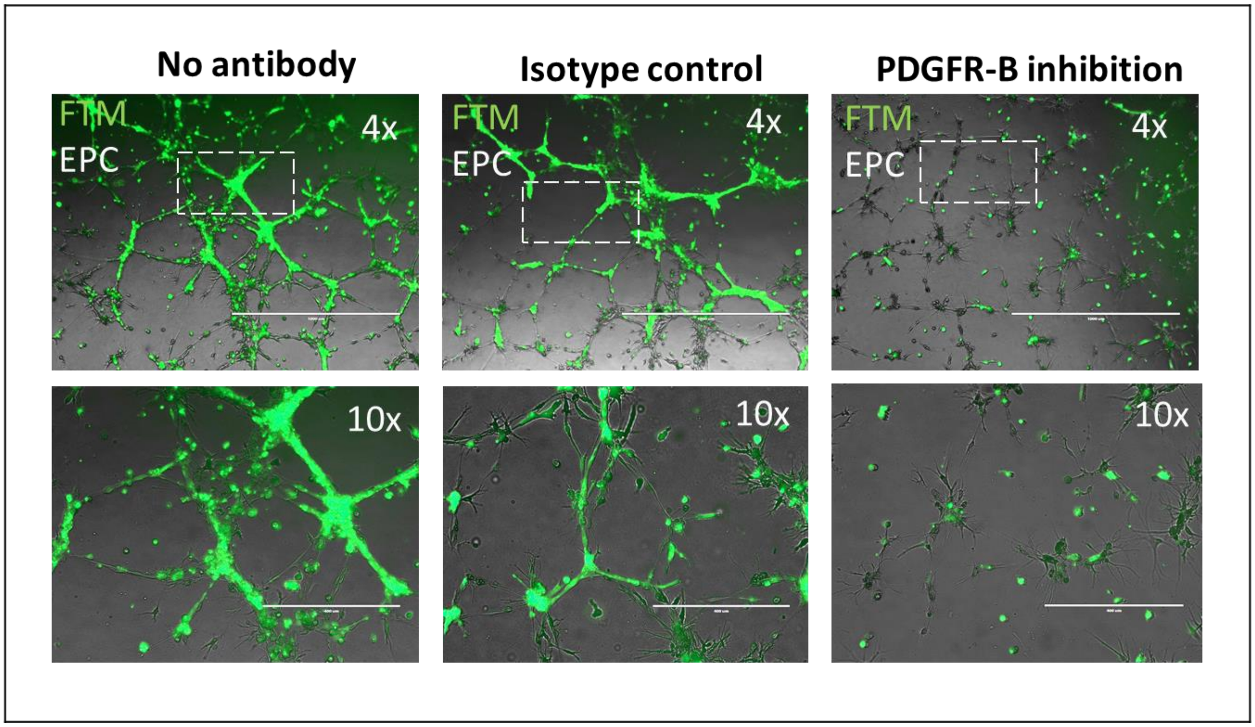

Supplementary Figure 15: FTM HUCPVC were incubated with primary PDGFR-β and isotype control antibodies for 20 minutes prior to ECFC co-culture. FTM HUCPVC incubated with anti-PDGFR-β significantly decreased homing to ECFC structures, while demonstrating small cell aggregates unable to support ECFC-mediated vascular formation. FTM HUCPVC from isotype control and no antibody treatment group significantly homed to ECFC-derived networks and localized adjunct at both nodes and tubular structures. Clear differences in tube formation properties were observed between isotype control and PDGFR-β neutralization treatment group. Low magnification scale bar: 1000 µm; high magnification scale bar: 400µm. N=3.

References

1. Gauthier-Fisher A, Szaraz P and Librach CL. Pericytes in the Umbilical Cord. *Adv Exp Med Biol*. 2019;1122:211-233.'10.1007/978-3-030-11093-2_12:' 10.1007/978-3-030-11093-2_12.
