# Supplementary material for: Combination human umbilical cord perivascular and endothelial colony forming cell therapy for ischemic cardiac injury
Source: NPJ Regen Med. 2023 Aug 25;8:45. doi: 10.1038/s41536-023-00321-3 (PMC10457300; doi:10.1038/s41536-023-00321-3)
Supplement: Supplementary file 2 — Reporting Summary [file 41536_2023_321_MOESM2_ESM.pdf]

nature portfolio

Corresponding author(s): Andree Gauthier-Fisher, Clifford L Librach

Last updated by author(s): 07/12/2023

Reporting Summary

Nature Portfolio wishes to improve the reproducibility of the work that we publish. This form provides structure for consistency and transparency in reporting. For further information on Nature Portfolio policies, see our Editorial Policies and the Editorial Policy Checklist.

Statistics

For all statistical analyses, confirm that the following items are present in the figure legend, table legend, main text, or Methods section.

n/a	Confirmed	
☐	☒	The exact sample size (*n*) for each experimental group/condition, given as a discrete number and unit of measurement
☐	☒	A statement on whether measurements were taken from distinct samples or whether the same sample was measured repeatedly
☐	☒	The statistical test(s) used AND whether they are one- or two-sided<br>*Only common tests should be described solely by name; describe more complex techniques in the Methods section.*
☒	☐	A description of all covariates tested
☐	☒	A description of any assumptions or corrections, such as tests of normality and adjustment for multiple comparisons
☐	☒	A full description of the statistical parameters including central tendency (e.g. means) or other basic estimates (e.g. regression coefficient) AND variation (e.g. standard deviation) or associated estimates of uncertainty (e.g. confidence intervals)
☒	☐	For null hypothesis testing, the test statistic (e.g. *F*, *t*, *r*) with confidence intervals, effect sizes, degrees of freedom and *P* value noted<br>*Give P values as exact values whenever suitable.*
☒	☐	For Bayesian analysis, information on the choice of priors and Markov chain Monte Carlo settings
☒	☐	For hierarchical and complex designs, identification of the appropriate level for tests and full reporting of outcomes
☒	☐	Estimates of effect sizes (e.g. Cohen's *d*, Pearson's *r*), indicating how they were calculated

Our web collection on statistics for biologists contains articles on many of the points above.

Software and code

Policy information about availability of computer code

Data collection	Cardiac pressure volume analysis was collected and extracted from PVAN 3.3 (Millar Instruments) software. Echocardiography measurements were extracted from GE Vivid 7 ultrasound system (GE Healthcare Canada) . In vitro tube formation analysis and matrigel plug vascular data was visualized and analyzed using open source Image J with the available angiogenesis plugin. Angiogenesis and inflammatory protein arrays were imaged and pixel intensity was calculated from HLA imaging software (purchased). Next generation sequencing NGS was performed using the Ion PGM sequencer, 400 flows (Invitrogen Life Technologies, Carlsbad, California, USA). Unaligned read files (FASTQ) were uploaded to Partek Flow (Partek, St Louis Missouri, United States) for bioinformatic analysis. Samples were trimmed for quantity, aligned to the rat genome (rn5) using Spliced Transcripts Alignment to a Reference (STAR) (v2.5.2)3 and subjected to post-alignment quality control. All unaligned reads were realigned to the human genome (hg19) using STAR (v2.5.2).
Data analysis	Data analysis and statistics for in vitro and in vivo studies was conducted using Graphpad Prism 9.

For manuscripts utilizing custom algorithms or software that are central to the research but not yet described in published literature, software must be made available to editors and reviewers. We strongly encourage code deposition in a community repository (e.g. GitHub). See the Nature Portfolio guidelines for submitting code & software for further information.

Data

Policy information about availability of data

All manuscripts must include a data availability statement. This statement should provide the following information, where applicable:

- Accession codes, unique identifiers, or web links for publicly available datasets
- A description of any restrictions on data availability
- For clinical datasets or third party data, please ensure that the statement adheres to our policy

The datasets generated during and/or analysed during the current study are available from the corresponding author on reasonable request.

Human research participants

Policy information about studies involving human research participants and Sex and Gender in Research.

Reporting on sex and gender	Mesenchymal stromal cell lines (umbilical cord perivascular cell lines) were expanded and studied from both male and female donors. The age of gestation ranged between week 8 and 10 of gestation for umbilical cords collected from first trimester pregnancies. Term human umbilical cord perivascular cells were collected and tested from both male and female donors. Rat endothelial colony forming cells were isolated and cultured from male Sprague dawley rats (8 weeks of age). Rats who were induced for myocardial infarction were all 8 week old, male nude (NIH-Foxn1rnu-Charles River) rats.
Population characteristics	N/A
Recruitment	N/A
Ethics oversight	University of Toronto Protocol # 28889

Note that full information on the approval of the study protocol must also be provided in the manuscript.

Field-specific reporting

Please select the one below that is the best fit for your research. If you are not sure, read the appropriate sections before making your selection.

☒ Life sciences ☐ Behavioural & social sciences ☐ Ecological, evolutionary & environmental sciences

For a reference copy of the document with all sections, see nature.com/documents/nr-reporting-summary-flat.pdf

Life sciences study design

All studies must disclose on these points even when the disclosure is negative.

Sample size	A power analysis was conducted to determine the sample size required to detect a change of at least 25% assuming a standard deviation of 15% for all experiments. For animal cardiac function analysis, a pilot study (n=4) was conducted to document the differences if any for ejection fraction and fractional shortening following therapy. Based on this, we enrolled 9 animals per treatment group. For in vitro experiments, pilot studies were conducted and due to drastically different observations per treatment group, 6 donors were included for analysis. Three donors were used for next generation sequencing analysis due to cost and feasibility. The same donors were used for both in vitro and in vivo experiments. Matrigel plug experiments included 4 animals per treatment group which was determined by a pilot study to detect at least a 25% difference between treatment groups.
Data exclusions	Two animals were excluded from both the five day and 14 days cardiac function analysis because a difference 30% in cardiac function from the other animals within the same cohort. No data was excluded for in vitro, next generation sequencing and matrigel plug assay experiments.
Replication	All experiments were replicated at least 3 times, with different donors, experimental setups and days of surgery.
Randomization	For cardiac function experiments, all animals were randomly assigned experimental groups based on the baseline criteria of having a fractional shortening measurement between 20-40%. For matrigel plug studies, each animal had 4 injected plugs. Each plug included a different treatment group to account for any variability between animals and experimental setups. For in vitro tube formation assays, all donors and treatments were tested at the same time to account for variability between culture conditions and variability innate from aortic rings, endothelial colony forming cells collected from varying animals.
Blinding	All experimental animals were coded and both the personnel conducting the surgeries/ cell injections, and conducting analysis were blinded. Codes were revealed following the completion of analysis. For in vitro studies, personal were not blinded between assay setup because same researcher cultured cell lines and conducted experiments. Following treatments at end points, experimental groups were coded and imaged by blinded personnel. Data analysis was conducted blinded. For matrigel plug assays, researcher injecting animals was blinded and analysis was conducted on coded groups. Next generation sequencing library preparation and analysis was all conducted blinded.

Behavioural & social sciences study design

All studies must disclose on these points even when the disclosure is negative.

Study description	N/A
Research sample	N/A
Sampling strategy	N/A
Data collection	N/A
Timing	N/A
Data exclusions	N/A
Non-participation	N/A
Randomization	N/A

Ecological, evolutionary & environmental sciences study design

All studies must disclose on these points even when the disclosure is negative.

Study description	N/A
Research sample	N/A
Sampling strategy	N/A
Data collection	N/A
Timing and spatial scale	N/A
Data exclusions	N/A
Reproducibility	N/A
Randomization	N/A
Blinding	*Describe the extent of blinding used during data acquisition and analysis. If blinding was not possible, describe why OR explain why blinding was not relevant to your study.*

Did the study involve field work? ☐ Yes ☒ No

Field work, collection and transport

Field conditions	N/A
Location	N/A
Access & import/export	N/A
Disturbance	N/A

Reporting for specific materials, systems and methods

We require information from authors about some types of materials, experimental systems and methods used in many studies. Here, indicate whether each material, system or method listed is relevant to your study. If you are not sure if a list item applies to your research, read the appropriate section before selecting a response.

Materials & experimental systems

n/a	Involved in the study
☐	☒ Antibodies
☐	☒ Eukaryotic cell lines
☒	☐ Palaeontology and archaeology
☐	☒ Animals and other organisms
☒	☐ Clinical data
☒	☐ Dual use research of concern

Methods

n/a	Involved in the study
☒	☐ ChIP-seq
☐	☒ Flow cytometry
☒	☐ MRI-based neuroimaging

Antibodies

Antibodies used

All antibodies and their respective catalog numbers are documented in the supplementary files.

Table 1: Primary antibodies for immunofluorescence staining
Antigen/Supplier/Dilution
Connexin-43 Abcam: ab11370 Goat1:200
Sarcomeric actinin Abcam: ab32575 Rabbit 1:200
PDGFR-β Abcam: ab62437 Rabbit 1:200
Isolectin-GS-IB4 Life Technologies:1-21412 -- 1:50
Thapsigargin Life Technologies: B-7487 Goat 1:200
Cleaved caspase Cell Signaling, 9661S Rabbit 1:200
DQ Gelatin Life Technologies - 1:50

Table 2. Secondary antibodies for immunofluorescence staining (all purchased from Thermofisher)
Anti-rat IgG AF594 1:500
Anti-mouse IgG AF488 1:500
Anti-rabbit IgG AF647 1:500
Anti-goat IgG A-11055 1:500
Anti-sheep IgG A-11015 1:500

Table 3. Flow cytometry human antibodies
Antigen/Supplier/Dilution
CD90-APC R&D systems: (#130-114-903) 1:40
CD105-APC R&D systems: (#130-098-778 1:40
CD146-FITC R&D systems:(#130-111-323) 1:40
HLA-G-FITC R&D systems (130-112-004) 1:40
PDGFR-β-APC R&D systems # FAB1263A 1:20

Table 4. Flow cytometry rat antibodies
Antigen/Supplier/Dilution
CD31-FITC R&D systems: (AF3628) 1:40
CD-34-FITC R&D systems: (AF6518) 1:40
CD133-PE Novus Biological (NB200-208) 1:40
CD38-APC Thermofisher (560846) 1:20
KDR-PE Novus Biological (NB-200-208) 1:20
CD146-FITC BD Sciences (560846) 1:20
VE-Cadherin-FITC BioUSA BS-0878R-A488 1:20
CD117-APC Tonbo Biosciences 20-1172-u025 1:20

Validation

All antibodies were optimized by our lab group for other studies before conducting this current study. If new antibodies were utilized, they were titrated and tested at different dilutions before using. Negative controls were included with secondary antibody for immunohistochemistry analysis to document background or baseline signal.

Eukaryotic cell lines

Policy information about cell lines and Sex and Gender in Research

Cell line source(s)

Human first trimester umbilical cord perivascular cells were collected from elected abortions between week 8-10 gestation. Both male and female donors were utilized for experiments. Cell lines were expanded and a true homogeneous population was confirmed by flow cytometry at passage 2 (testing for mesenchymal stromal cells and pericyte antigens). Cells lines were utilized at passage 6 for all experiments and confirmed for phenotype by flow cytometry before testing. Human term umbilical cord were collected from term pregnancies. The same protocols mentioned above were implemented. Endothelial colony forming cells were collected from the femur and tibia of 8 week old sprauge dawley rats. Cells were cultured in endothelial growth medium, where primary cells were cultured for 14 days and processed for flow cytometry. A panel of endothelial and progenitor markers were analyzed before conducting experiments. These cells were not passaged and used fresh after the 14 days of culture.

Authentication	All cell lines were authenticated before experiments by flow cytometry. If human umbilical cord perivascular cell lines met the criteria for mesenchymal stromal cells and pericyte antigens, they were processed for experiments. The same was done for endothelial colony forming cells.
Mycoplasma contamination	All cell lines tested negative for mycoplasma contamination.
Commonly misidentified lines (See ICLAC register)	Not applicable.

Palaeontology and Archaeology

Specimen provenance	N/A
Specimen deposition	N/A
Dating methods	N/A

☐ Tick this box to confirm that the raw and calibrated dates are available in the paper or in Supplementary Information.

Ethics oversight	N/A

Note that full information on the approval of the study protocol must also be provided in the manuscript.

Animals and other research organisms

Policy information about studies involving animals; ARRIVE guidelines recommended for reporting animal research, and Sex and Gender in Research

Laboratory animals	For matrigel plug assays, FoxN1nu 8-week-old male mice were utilized. For cardiac function experiments, 8-week-old male nude rats (NIH-Foxn1rnu-Charles River) were utilized. For endothelial colony forming cells, 8-week-old male sprague dawley rats were utilized.
Wild animals	Not applicable.
Reporting on sex	All mice and rats used in these experiments were male. The therapeutics cells tested from human umbilical cords included both male and female donors. Since the presented experiments have two components (the donor and recipient) which could increase the variability beyond our control, we chose to include both male and female genders for testing cell therapeutics while keeping the recipients of treatment the same sex (male).
Field-collected samples	Not applicable.
Ethics oversight	All studies were approved by the ethics committee at our institute. Research ethics board approval was obtained for the collection of first trimester (8-10 weeks of gestation) from elective pregnancy termination and full-term human umbilical cords (REB #28889, University of Toronto, Canada). Written informed consent was obtained for each sample. Term newborn cords were collected through a third party (Lifeline Stem Cell). All animal procedures were conducted and reported according to ARRIVE guidelines and approved by the Animal Care Committee of the University Health Network (Toronto, Canada). All studies were performed with institutional research ethics board approval (AUP 4276, University of Toronto, Toronto, Canada).

Note that full information on the approval of the study protocol must also be provided in the manuscript.

Clinical data

Policy information about clinical studies

All manuscripts should comply with the ICMJE guidelines for publication of clinical research and a completed CONSORT checklist must be included with all submissions.

Clinical trial registration	N/A
Study protocol	N/A
Data collection	N/A
Outcomes	N/A

Dual use research of concern

Policy information about dual use research of concern

Hazards

Could the accidental, deliberate or reckless misuse of agents or technologies generated in the work, or the application of information presented in the manuscript, pose a threat to:

No	Yes	
☒	☐	Public health
☒	☐	National security
☒	☐	Crops and/or livestock
☒	☐	Ecosystems
☒	☐	Any other significant area

Experiments of concern

Does the work involve any of these experiments of concern:

No	Yes	
☒	☐	Demonstrate how to render a vaccine ineffective
☒	☐	Confer resistance to therapeutically useful antibiotics or antiviral agents
☒	☐	Enhance the virulence of a pathogen or render a nonpathogen virulent
☒	☐	Increase transmissibility of a pathogen
☒	☐	Alter the host range of a pathogen
☒	☐	Enable evasion of diagnostic/detection modalities
☒	☐	Enable the weaponization of a biological agent or toxin
☒	☐	Any other potentially harmful combination of experiments and agents

ChIP-seq

Data deposition

☐ Confirm that both raw and final processed data have been deposited in a public database such as GEO.

☐ Confirm that you have deposited or provided access to graph files (e.g. BED files) for the called peaks.

Data access links<br>*May remain private before publication.*	*For "Initial submission" or "Revised version" documents, provide reviewer access links. For your "Final submission" document, provide a link to the deposited data.*
Files in database submission	*Provide a list of all files available in the database submission.*
Genome browser session<br>(e.g. UCSC)	*Provide a link to an anonymized genome browser session for "Initial submission" and "Revised version" documents only, to enable peer review. Write "no longer applicable" for "Final submission" documents.*

Methodology

Replicates	*Describe the experimental replicates, specifying number, type and replicate agreement.*
Sequencing depth	*Describe the sequencing depth for each experiment, providing the total number of reads, uniquely mapped reads, length of reads and whether they were paired- or single-end.*
Antibodies	*Describe the antibodies used for the ChIP-seq experiments; as applicable, provide supplier name, catalog number, clone name, and lot number.*
Peak calling parameters	*Specify the command line program and parameters used for read mapping and peak calling, including the ChIP, control and index files used.*
Data quality	*Describe the methods used to ensure data quality in full detail, including how many peaks are at FDR 5% and above 5-fold enrichment.*
Software	*Describe the software used to collect and analyze the ChIP-seq data. For custom code that has been deposited into a community repository, provide accession details.*

Flow Cytometry

Plots

Confirm that:

☒ The axis labels state the marker and fluorochrome used (e.g. CD4-FITC).

☒ The axis scales are clearly visible. Include numbers along axes only for bottom left plot of group (a 'group' is an analysis of identical markers).

☒ All plots are contour plots with outliers or pseudocolor plots.

☒ A numerical value for number of cells or percentage (with statistics) is provided.

Methodology

Sample preparation

For flow cytometry (FC) and fluorescence associated- cell sorting (FACS) of human umbilical cord perivascular cells, cell cultures were dissociated (TrypleE) and counted by an automatic cell counter. Cell suspensions were incubated with fluorophore conjugated primary antibodies, according to the provider's description. For most antibodies, 50,000 cells were incubated with 2.5µl of antibody for 20 minutes at 4ºC. FC analysis was performed using either a MACQuant analyzer (Miltenyi Biotec; Create Fertility Centre, Toronto) or digital (LSR II, Canto II, BD; UHN SickKids Flow Cytometry Facility, Toronto) analytical cytometers. FACS was performed using digital cell sorters (MoFlo Astrios, Aria II, UHN SickKids Flow Cytometry Facility, Toronto). Human specific TRA-1-85+ sorted cells were collected in lysis buffer (Qiagen) for qPCR and next generation sequencing (NGS). Fluorescence signals were gated based on unstained populations and gates were set at 10^1 fluorescence intensity decades. Mean fluorescence intensities were used for analysis. Antibodies used for identifying HUCPVC phenotypes included CD90-APC (#130-114-903), CD105-APC (#130-098-778), CD146-FITC (#130-111-323), HLA-G-FITC (130-112-004) at a 1:40 dilution (Miltenyi) and PDGB-R-APC (R&D # FAB1263A) at 1:20 dilution (Data Supplement, Table 3).

For flow cytometry (FC) and fluorescence-associated cell sorting (FACS) of endothelial colony forming cells, cell cultures were dissociated (TrypleE) and counted using an automated cell counter. Cell suspensions were incubated with fluorophore conjugated primary antibodies according to the providers description. For most antibodies, 50,000 cells were incubated with 2.5µl of antibody for 20 minutes at 4ºC. FC analysis was performed using the MACQuant analyzer (Miltenyi Biotec; Create Fertility Centre, Toronto). FACS was performed using digital cell sorters (MoFlo Astrios, Aria II, UHN SickKids Flow Cytometry Facility, Toronto). Fluorescence signals were gated based on unstained populations. Gates were set at 10^1 fluorescence intensity decades. Antibodies for ECFC FC were as follows: CD31 (AF3628 R&D-FITC), CD34 (AF6518 R&D-FITC), CD133 (NB120-16518 Novus Biologicals-PE), CD38 (50-0380-80 Thermofisher-APC), KDR (NB200-208 Novus Biologicals-PE), CD146 (560846 BD sciences-FITC), VE-Cadherin (BS-0878R-A488 BioUSA-FITC) and CD117 (20-1172-u025- Tonbo Science-APC). All antibody titrations were performed and used at a concentration of 1:20 (Data Supplement, Table 4 and Figure II and III).

Instrument

Flow cytometry analysis was performed using the MACQuant analyzer (Miltenyi Biotec; Create Fertility Centre, Toronto). FACS was performed using digital cell sorters (MoFlo Astrios, Aria II, UHN SickKids Flow Cytometry Facility, Toronto).

Software

Flow cytomtry data was analyzed and extracted from flowJo,LLC software.

Cell population abundance

At least 10,000 cells from preliminary gate were analyzed.

Gating strategy

Fluorescence signals were gated based on unstained populations. Gates were set at 10^1 fluorescence intensity decades. First, a clear dense population was selected based on FSC and SSC parameters on unstained cells. A gate was established based on unstained populations for each marker and associated fluorescence signal. At least 10,000 events were analyzed for each selected gate. A specific antibody was not selected for preliminary analysis to avoid biases in analysis, since all cell lines were analyzed and tested by flow cytometry prior to experiments.

☒ Tick this box to confirm that a figure exemplifying the gating strategy is provided in the Supplementary Information.

Magnetic resonance imaging

Experimental design

Design type

Indicate task or resting state; event-related or block design.

Design specifications

Specify the number of blocks, trials or experimental units per session and/or subject, and specify the length of each trial or block (if trials are blocked) and interval between trials.

Behavioral performance measures

State number and/or type of variables recorded (e.g. correct button press, response time) and what statistics were used to establish that the subjects were performing the task as expected (e.g. mean, range, and/or standard deviation across subjects).

Acquisition

Imaging type(s) — *Specify: functional, structural, diffusion, perfusion.*

Field strength — *Specify in Tesla*

Sequence & imaging parameters — *Specify the pulse sequence type (gradient echo, spin echo, etc.), imaging type (EPI, spiral, etc.), field of view, matrix size, slice thickness, orientation and TE/TR/flip angle.*

Area of acquisition — *State whether a whole brain scan was used OR define the area of acquisition, describing how the region was determined.*

Diffusion MRI ☐ Used ☐ Not used

Preprocessing

Preprocessing software — *Provide detail on software version and revision number and on specific parameters (model/functions, brain extraction, segmentation, smoothing kernel size, etc.).*

Normalization — *If data were normalized/standardized, describe the approach(es): specify linear or non-linear and define image types used for transformation OR indicate that data were not normalized and explain rationale for lack of normalization.*

Normalization template — *Describe the template used for normalization/transformation, specifying subject space or group standardized space (e.g. original Talairach, MNI305, ICBM152) OR indicate that the data were not normalized.*

Noise and artifact removal — *Describe your procedure(s) for artifact and structured noise removal, specifying motion parameters, tissue signals and physiological signals (heart rate, respiration).*

Volume censoring — *Define your software and/or method and criteria for volume censoring, and state the extent of such censoring.*

Statistical modeling & inference

Model type and settings — *Specify type (mass univariate, multivariate, RSA, predictive, etc.) and describe essential details of the model at the first and second levels (e.g. fixed, random or mixed effects; drift or auto-correlation).*

Effect(s) tested — *Define precise effect in terms of the task or stimulus conditions instead of psychological concepts and indicate whether ANOVA or factorial designs were used.*

Specify type of analysis: ☐ Whole brain ☐ ROI-based ☐ Both

Statistic type for inference (See Eklund et al. 2016) — *Specify voxel-wise or cluster-wise and report all relevant parameters for cluster-wise methods.*

Correction — *Describe the type of correction and how it is obtained for multiple comparisons (e.g. FWE, FDR, permutation or Monte Carlo).*

Models & analysis

n/a	Involved in the study
☐	☐ Functional and/or effective connectivity
☐	☐ Graph analysis
☐	☐ Multivariate modeling or predictive analysis

Functional and/or effective connectivity — *Report the measures of dependence used and the model details (e.g. Pearson correlation, partial correlation, mutual information).*

Graph analysis — *Report the dependent variable and connectivity measure, specifying weighted graph or binarized graph, subject- or group-level, and the global and/or node summaries used (e.g. clustering coefficient, efficiency, etc.).*

Multivariate modeling and predictive analysis — *Specify independent variables, features extraction and dimension reduction, model, training and evaluation metrics.*
